# Multivariate functional group sparse regression: Functional predictor selection

**DOI:** 10.1371/journal.pone.0265940

**Published:** 2022-04-07

**Authors:** Ali Mahzarnia, Jun Song

**Affiliations:** 1 Department of Mathematics and Statistics, University of North Carolina at Charlotte, Charlotte, NC, United States of America; 2 Department of Statistic, Korea University, Seoul, South Korea; University of Nevada, Reno, UNITED STATES

## Abstract

In this paper, we propose methods for functional predictor selection and the estimation of smooth functional coefficients simultaneously in a scalar-on-function regression problem under a high-dimensional multivariate functional data setting. In particular, we develop two methods for functional group-sparse regression under a generic Hilbert space of infinite dimension. We show the convergence of algorithms and the consistency of the estimation and the selection (oracle property) under infinite-dimensional Hilbert spaces. Simulation studies show the effectiveness of the methods in both the selection and the estimation of functional coefficients. The applications to functional magnetic resonance imaging (fMRI) reveal the regions of the human brain related to ADHD and IQ.

## 1 Introduction

In the past decades, functional data analysis (FDA) has received great attention in which an entire function is treated as a single observation. [1] introduced a general framework of FDA. The major challenges of FDA is that the methods are based on an infinite-dimensional space so that it is difficult to extend the traditional statistical methods in a straightforward way. Many other researchers tackled the problems and investigated the estimation and inference methods of functional data. For example, [2] and [3] developed functional linear regression model for sparse longitudinal data analysis. [4] developed weighted functional linear Cox regression model. [5, 6] further summarized methods and inferences of FDA. More recently, FDA has been extended to multivariate functional data that can deal with multiple functions as a single observation. See [7, 8]. These development of FDA has been applied to various fields of study. For example, tumor physiology data, orthosis data, and metabolite progesterone curves applied FDA [9]. In the field of earth science, [10] uses the meteorology functional data of recorded precipitation, wind speed, and temperature during the days of a month. Although FDA has received a lot of attention in recent decades, the sparseness of functional predictors in the multivariate model has not been studied well compared to the univariate case. Hence, we aim to develop theories and algorithms for the sparse functional regression methods with functional predictor selection when we have scalar data as response values and high-dimensional multivariate functional data as predictors.

Under the multivariate setting, numerous sparse models have been studied with the introduction of *L*_1_-penalty. Least absolute shrinkage and selection operator (LASSO) introduces a penalty term to the least square cost function which performs both variable selection and shrinkage [11]. The LASSO-type penalty, such as the Elastic Net [12], the smoothly clipped absolute deviation (SCAD) [13], their modifications (the adaptive LASSO [14] and the adaptive Elastic Net [15]) are developed to overcome the lack of theoretical support and the practical limitations of the LASSO such as the saturation. These methods were developed to overcome the challenges and enjoy asymptotic properties when the sample size increases, such as the estimation consistency and the selection consistency, also known as the oracle property.

Recently, the sparse models have been extended to the functional data. Initially, a majority of the literature seeks the sparseness of the time domain. Examples include [16] and related articles for univariate functional data and [17] for multivariate functional data. On the other hand, [18] proposed a model considering the sparseness in the functional predictors under the multivariate functional data setting. In particular, they introduced a model based on the least absolute deviation (LAD) and the group LASSO in the presence of outliers in functional predictors and responses. Its numerical examples and data application show the effectiveness in practice, but theoretical properties and detailed algorithms have not been explored. More recently, [19] proposed multivariate functional principal component analysis considering the sparsity between functional predictors, but it is an unsupervised version. To this end, we develop methods for the scalar-on-function regression model which allows sparseness of the functional predictors and the simultaneous estimation of the smooth functional coefficients. To implement it with the actual data, we derive two algorithms for each of the optimization problems. Finally, we show both the functional predictor selection consistency and the estimation consistency.

**Fig 1:**
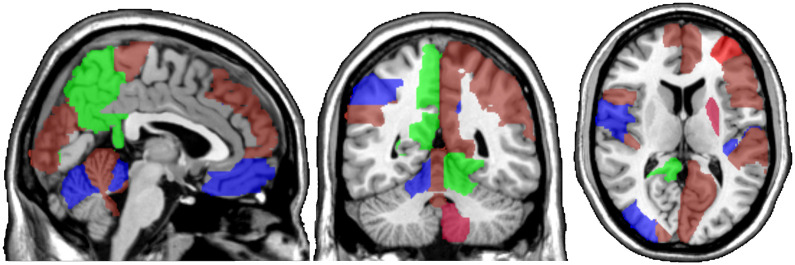
The regions of interests, the BOLD activities of which correlate the most with the ADHD score variability in a sample of subjects and achieve the lowest prediction error. The regions associated with ADHD are colored red, those associated with ADHD Hyper/Impulsive are blue, and the ones associated with ADHD Inattentive are colored green.

One motivating example for our methods is the application to functional magnetic resonance imaging (fMRI). The dataset consists of the functional signals of the brain activities measured by blood-oxygen-level-dependent (BOLD), which detects hemodynamic changes based on the metabolic demands followed by neural activities. There are pre-specified regions of the brain, and the BOLD signals associated with multiple voxels in each region are integrated into one signal for that region. Thus, the fMRI data are considered to be multivariate functional data in which each functional predictor represents the signals from a region of the brain. In Section 8, we regress the ADHD index to the regional BOLD activities of the fMRI of the human subjects. There are 116 regions of the brain in the data, and our methods reduce the regions to 41 regions with significantly lower errors than the linear functional regression. Fig 1 displays the regions of the brain’s atlas that are identified by our method. It shows that the methods simplify the data analysis and provide clear representation while keeping the crucial information. The analysis shows that there is an urgent need for new methods in the fields of medical and life sciences as well as other related areas. The following quote from [20] further motivates us to study the applications of the sparse multivariate functional regression in the field of fMRI.

“Think of the challenge of the fMRI with the analogous situation one would have if, when flying over a city at night, an attempt is made to determine the city activities in detail by simply observing where the lights are on. The information is extremely sparse, but with time, specific inferences can be drawn.”

— Peter A. Bandettini, *fMRI*, 2020

The rest of the paper is organized as follows. In Section 2, we illustrate the general framework of our methods along with the notations used in this paper. In Section 3, we describe the model and the optimization problem that we consider. Then, we develop an explicit solution to the optimization problem and illustrate a detailed procedure using alternating direction method of multipliers (ADMM) in Section 4. We also derive another algorithm, called groupwise-majorization-descent (GMD), along with the strong rule for faster computation in Section 5. In Section 6, we develop asymptotic results, including the consistency of our methods and the oracle property. In Section 7, we show the effectiveness of the methods by conducting simulation studies. In Section 8, we apply the methods to a resting–state fMRI dataset. Concluding discussions are made in section 9. Finally, the supplementary materials include all of the proofs and the list of the regions of the brain associated with ADHD and IQ scores. We created an R package MFSGrp for the computation, and it is available at https://github.com/Ali-Mahzarnia/MFSGrp.

## 2 Preliminary and notation

Let (Ω,F,P) be a probability space where Ω is a sample space, F is a *σ*-field and *P* be a probability measure on it. Let *T*_*j*_ be a compact set in $R^{d_{j}}$ for *j* = 1, …, *p*. Let $H^{1},\ldots ,H^{p}$ be separable Hilbert spaces of functions from *T*_*j*_ to R with an inner product ${\langle \cdot ,\cdot \rangle }_{H^{j}}$. Let $H=H^{1}\times \cdots \times H^{p}$ be endowed with the inner product

$$\begin{array}{c} {\left\langle f,g\right\rangle }_{H}={\left\langle f^{1},g^{1}\right\rangle }_{H^{1}}+\ldots +{\left\langle f^{p},g^{p}\right\rangle }_{H^{p}}, \end{array}$$

for any $f={\left(f^{1},\ldots ,f^{p}\right)}^{⊤}\in H$, and $g={\left(g^{1},\ldots ,g^{p}\right)}^{⊤}\in H$. Then, H is also a separable Hilbert space. Let X:Ω→H be a measurable function with respect to F/B where B is the Borel *σ*-field generated by open sets in H.

Let *X* be a random element in H. If ${E\|X\|}_{H}<\infty$; then, the linear functional $f\mapsto E{\langle f,X\rangle }_{H}$ is bounded. By Riesz’s representation theorem, there is a unique element in H, say *μ*_*X*_, such that ${\langle \mu_{X},f\rangle }_{H}=E{\langle f,X\rangle }_{H}$ for any f∈H. For more details about the fundamentals of the functional data, see [21]. We call *μ*_*X*_ the mean element of *X* or expectation of *X*. If we can further assume ${E\|X\|}_{H}^{2}<\infty$, the operator H→H,

$$\begin{array}{c} \Gamma_{XX}=E\left[\left\{X-E\left(X\right)\right\}⊗\left\{X-E\left(X\right)\right\}\right], \end{array}$$ (1)

exists and is a Hilbert-Schmidt operator, where ⊗ indicates a tensor product computed in a way that for x,y,z∈H, $\left(x⊗y\right)\left(z\right)={\langle y,z\rangle }_{H}x$. [22].

Let *Y* be a random element in $H_{Y}$. Subsequently, we can define the covariance operator between *X* and *Y* by

$$\begin{array}{c} \Gamma_{YX}=E\left[\left\{Y-E\left(Y\right)\right\}⊗\left\{X-E\left(X\right)\right\}\right], \end{array}$$

which maps from $H_{X}$ to $H_{Y}$. Γ_*XY*_ can be similarly defined. For convenience, throughout this paper, we assume that *E*(*X*) = 0 and *E*(*Y*) = 0 without loss of generality. Hence, the regression model is

$$\begin{array}{c} Y={\left\langle X,\beta \right\rangle }_{H}+\epsilon , \end{array}$$

where β∈H is the unknown coefficient function, and *ϵ* is an error term which is a mean zero random variable and independent of *X*. Consider *Y* as a scalar random variable. We can rewrite *β*(·) = (*β*^1^(·), …, *β*^*p*^(·)) and

$$\begin{array}{c} {\left\langle X,\beta \right\rangle }_{H}=\sum_{j=1}^{p}{\left\langle X^{j},\beta^{j}\right\rangle }_{H^{j}}. \end{array}$$

## 3 Model description

We are interested in the situations where the predictors are multivariate functions but only a few functional predictors affecting the response. i.e., a random variable *Y* and random functions $X^{j}\in H^{j}$ have the following relation,

$$\begin{array}{c} Y=\sum_{j\in J}{\left\langle X^{j},\beta^{j}\right\rangle }_{H^{j}}+\epsilon , \end{array}$$ (2)

where *J* ⊆ {1, …, *p*} is an unknown active set of indices involved in this regression model, and *ϵ* is a mean zero error term that is independent of *X*.

Assume that we have a random sample of size *n* from the model (2). To estimate *β* and the active set *J*, we propose the following objective function.

$$\begin{array}{c} L\left(\beta ;\lambda_{1n}\right)=\frac{1}{2}E_{n}{\left(Y-{\left\langle X,\beta \right\rangle }_{H}\right)}^{2}+\lambda_{1n}\sum_{j=1}^{p}{\left\|\beta^{j}\right\|}_{H^{j}},\;\beta \in H, \end{array}$$ (3)

where *E*_*n*_ is the expectation with the empirical distribution. We added the group-lasso type penalty so that each group includes one functional component in the infinite–dimensional Hilbert space, $H^{j}$, *j* = 1, …, *p*. Note that the norm in the penalty term is *L*_2_-norm which makes the objective function convex. In addition, we propose an alternative objective function to gain a more stable solution path.

$$\begin{array}{c} L\left(\beta ;\lambda_{1n},\lambda_{2n}\right)=\frac{1}{2}E_{n}{\left(Y-{\left\langle X,\beta \right\rangle }_{H}\right)}^{2}+\lambda_{1n}\sum_{j=1}^{p}\|\beta^{j}{\|}_{H^{j}}+\lambda_{2n}\sum_{j=1}^{p}{\left\|\beta^{j}\right\|}_{H^{j}}^{2},\;\beta \in H. \end{array}$$ (4)

The quadratic term allows us to have a stable solution path. To be specific, the quadratic term makes the objective function strongly convex so that it gives us the unique solution. It is similar to the Elastic Net proposed by [12], but it is different in that the norm in the first penalty term uses *L*_2_-norm, and both the two penalties are applied group-wisely. The group-wise second penalty also gives us a huge computational advantage.

Furthermore, we also consider the smoothing penalty of the functional coefficients β∈H by adding the term, $\lambda_{3n}{\left\|\beta^{\prime \prime }\right\|}_{H}^{2}$ to the objective functions, (3) and (4). This additional penalty ‖*β*″‖, the *L*_2_-norm of the second derivative, measures the curvature of the coefficient function. Thus, by imposing the penalty on it, we can control the roughness or smoothness of a curve. It allows us to estimate smooth functional coefficients and to select the functional predictors simultaneously. In addition, it provides a better interpretation of the functional coefficients in this linear functional regression model.

## 4 Estimation: ADMM

In this section, we develop the algorithm for solving the optimization problems introduced in Section 3 via ADMM, one that is popularly used in a general convex optimization problem. See [23]. Consider the following optimization problem.

$$\begin{array}{cc} \text{arg}\;\underset{\beta ,\gamma }{\text{min}}\; & f(\beta )+g(\gamma )\\ s.t.\; & \beta -\gamma =0, \end{array}$$ (5)

where *γ* is duplicate variable in H, $f\left(\beta \right)=\frac{1}{2}E_{n}{\left(Y-{\langle X,\beta \rangle }_{H}\right)}^{2}$, and $g\left(\gamma \right)=\lambda \sum_{j=1}^{p}{\left\|\gamma^{j}\right\|}_{H^{j}}$. Blocks *γ*^*j*^ are associated with their counterparts’ blocks *β*^*j*^. The augmented Lagrangian with its parameter *ρ* > 0 is

$$\begin{array}{c} L_{\rho }\left(\beta ,\gamma ,\eta \right)=f\left(\beta \right)+g\left(\gamma \right)+{\left\langle \eta ,\beta -\gamma \right\rangle }_{H}+\frac{\rho }{2}{\left\|\beta -\gamma \right\|}_{H}^{2}, \end{array}$$ (6)

where the Lagrangian multiplier is η∈H. The ADMM update rules are

$$\begin{array}{c} \begin{array}{c} \beta^{\text{new}}≔\text{arg}\;\underset{\beta }{\text{min}}\;L_{\rho }\left(\beta ,\gamma ,\eta \right)\\ \gamma^{\text{new}}≔\text{arg}\;\underset{\gamma }{\text{min}}\;L_{\rho }\left(\beta^{\text{new}},\gamma ,\eta \right)\\ \eta^{\text{new}}≔\eta +\rho (\beta^{\text{new}}-\gamma^{\text{new}}). \end{array} \end{array}$$ (7)

For computational convenience, it is a usual practice to consider the scaled dual parameter of the ADMM. Let $u=\frac{1}{\rho }\eta$. It is straightforward to verify that the update rules (7) with a scaled dual parameter are equivalent to

$$\begin{array}{c} \begin{array}{c} \beta^{\text{new}}≔\text{arg}\;\underset{\beta }{\text{min}}(f\left(\beta \right)+\frac{\rho }{2}{\left\|\beta -\gamma +U\right\|}_{H}^{2})\\ \gamma^{\text{new}}≔\text{arg}\;\underset{\gamma }{\text{min}}(g\left(\gamma \right)+\frac{\rho }{2}{\left\|\beta^{\text{new}}-\gamma +U\right\|}_{H}^{2})\\ U^{\text{new}}≔U+\beta^{\text{new}}-\gamma^{\text{new}}. \end{array} \end{array}$$ (8)

### 4.1 Coordinate representation of functional data

Our method is based on the basis-expansion approach to the functional data. Suppose that we have *n* random copies from the model (2) denoted by (*X*_1_, *Y*_1_), …, (*X*_*n*_, *Y*_*n*_) and we observe $X_{i}^{j}$ on $\left\{t_{i1}^{j},\ldots ,t_{ia_{i}^{j}}^{j}\right\}$ for each *i* = 1, …, *n* and *j* = 1, …, *p*. It allows us the functional data to be observed at a different set of time points for each subject and functional covariate. Furthermore, it corresponds to the population model presented in Section 3.

At the sample level, we assume that $H^{j}$ is spanned by a given set of basis functions, $B^{j}=\left\{b_{1}^{j},\ldots ,b_{m_{j}}^{j}\right\}$. It provides the flexibility of the structure of the basis system and also corresponds to the population setting in Section 3. Thus, for any $f\in H^{j}$, there exists a unique vector $a\in R^{m_{j}}$ such that $f\left(\cdot \right)=\sum_{k=1}^{m_{j}}a_{k}b_{k}^{j}\left(\cdot \right)$. We call the vector *a*, the coordinate of *f* and denote it ${\left[f\right]}_{B^{j}}$. We also assume that $H^{j}$ is constructed with the *L*_2_-inner product with respect to the Lebesgue measure,

$$\begin{array}{c} {\left\langle f,g\right\rangle }_{H^{j}}=\int_{T_{j}}f\left(t\right)g\left(t\right)dt,\;\text{for}\;\text{any}\;f,g\in H^{j}. \end{array}$$

Let *G*_*j*_ be *m*_*j*_ × *m*_*j*_ matrix whose (*i*, *k*)-th entry is ${\langle b_{i}^{j},b_{k}^{j}\rangle }_{H^{j}}=\int_{T_{j}}b_{i}^{j}\left(t\right)b_{k}^{j}\left(t\right)dt$, and let *G* be *M* × *M* block-diagonal matrix whose *j*-th block is *G*_*j*_ where $M=\sum_{j=1}^{p}m_{j}$. Consequently, for any f,g∈H,

$$\begin{array}{c} {\left\langle f,g\right\rangle }_{H}=\sum_{j=1}^{p}\sum_{i=1}^{m_{j}}\sum_{k=1}^{m_{j}}{\left({\left[f^{j}\right]}_{B^{j}}\right)}_{i}{\left({\left[g^{j}\right]}_{B^{j}}\right)}_{k}{\left\langle b_{i}^{j},b_{k}^{j}\right\rangle }_{H^{j}}=\sum_{j=1}^{p}{\left[f^{j}\right]}_{B^{j}}^{⊤}G^{j}{\left[g^{j}\right]}_{B^{j}}={\left[f\right]}_{B}^{⊤}G{\left[g\right]}_{B}, \end{array}$$

where ${\left[f\right]}_{B},{\left[g\right]}_{B}$ are the $R^{M}$-dimensional vectors obtained by stacking ${\left[f^{j}\right]}_{B^{j}}$ and ${\left[g^{j}\right]}_{B^{j}}$ respectively. We use the basis-expansion approach for each functional covariate $X_{i}^{j}$ for *i* = 1, …, *n* and *j* = 1, …, *p*, which is also used in [24, 25]. Without loss of generality, we assume *m* = *m*_1_ = ⋯ = *m*_*p*_ and *M* = *pm*.

Suppose that *A* is a linear operator from $H_{1}$ to $H_{2}$ in which the basis for $H_{1}$ is $B=\{b_{1},\ldots ,b_{m}\}$ and the basis for $H_{2}$ is $C=\{c_{1},\ldots ,c_{k}\}$. Then, we define the coordinate representation of the operator *A* to be *k* × *m* matrix, say ${}_{C}{\left[A\right]}_{B}$, whose (*i*, *j*)-th entry is ${\left({\left[Ab_{j}\right]}_{C}\right)}_{i}$. It can be easily shown that ${}_{C}{\left[Ax\right]}_{B}={}_{C}{\left[A\right]}_{B}{\left[x\right]}_{B}$ for any $x\in H_{1}$. For notational convenience, if the basis system is obvious in the context, we remove the subscripts of the coordinate representation throughout this paper. The following lemma provides a further simplification for easy computations.

**Lemma 1** Let $Q=I-n^{-1}1_{n}1_{n}^{⊤}$. *Let* ${\left[X_{1:n}\right]}_{B}$ *be the pm* × *n matrix, the k-th column of which is* ${\left[X_{k}\right]}_{B}$. *Then*

$$\begin{array}{c} {}_{B}{\left[{\hat{\Gamma }}_{XX}\right]}_{B}=n^{-1}{\left[X_{1:n}\right]}_{B}Q{\left[X_{1:n}\right]}_{B}^{⊤}G=n^{-1}{\left[{\tilde{X}}_{1:n}\right]}_{B}{\left[{\tilde{X}}_{1:n}\right]}_{B}^{⊤}G, \end{array}$$

*where* $\left[{\tilde{X}}_{1:n}\right]{}_{B}={\left[X_{1:n}\right]}_{B}Q$. *In addition, let Y be the n-dimensional vector, the elements of which are the observations Y*_1_, …, *Y*_*n*_. *Then*

$$\begin{array}{c} \left[{\hat{\Gamma }}_{YX}\right]=n^{-1}Y^{⊤}{\left[{\tilde{X}}_{1:n}\right]}_{B}^{⊤}G. \end{array}$$

### 4.2 Orthogonalization

To achieve computational efficiency, we orthonormalize the basis system via Karhunen-Loève expansion of the covariance operator of each of the functional predictors. For each *j* = 1, …, *p*, define Γ_*jj*_ to be the covariance operator of *X*^*j*^. Consequently, we have the following lemma.

**Lemma 2** *Let* $\left(\lambda_{1}^{j},v_{1}^{j}\right),\ldots ,\left(\lambda_{m}^{j},v_{m}^{j}\right)$ *be the pairs of eigenvalues and vectors of* $n^{-1}{\left(G^{j}\right)}^{1/2}{\left[{\tilde{X}}_{1:n}^{j}\right]}_{B^{j}}{\left[{\tilde{X}}_{1:n}^{j}\right]}_{B^{j}}^{⊤}{\left(G^{j}\right)}^{1/2}$ *with* $\lambda_{1}^{j}\geq \ldots \geq \lambda_{m}^{j}$ , *and let* ${\left[\phi_{k}^{j}\right]}_{B^{j}}={\left(G^{j}\right)}^{-1/2}v_{k}^{j}$ *for k* = 1, …, *m*. *Then, the Karhunen-Loève expansion of* ${\hat{\Gamma }}_{jj}$ *is*

$$\begin{array}{c} {\hat{\Gamma }}_{jj}=\sum_{k=1}^{m}\lambda_{k}^{j}\phi_{k}^{j}⊗\phi_{k}^{j}. \end{array}$$

Define a *m* × *m* matrix

$$\begin{array}{c} \Phi^{j}=(\begin{array}{ccc} {\left[\phi_{1}^{j}\right]}_{B^{j}} & \cdots & {\left[\phi_{m}^{j}\right]}_{B^{j}} \end{array}). \end{array}$$

Since $\phi_{m}^{j}$’s are the eigenfunctions of a self-adjoint operator, they are orthonormal. Thus, for any $x\in H^{j}$,

$$\begin{array}{cc} x(\cdot ) & =\sum_{k=1}^{m}{\left\langle x,\phi_{k}^{j}\right\rangle }_{H^{j}}\phi_{k}^{j}\left(\cdot \right)\\ & =\sum_{k=1}^{m}{\left[x\right]}_{B^{j}}^{⊤}G^{j}{\left[\phi_{k}^{j}\right]}_{B^{j}}\phi_{k}^{j}\left(\cdot \right). \end{array}$$

Define $C^{j}=\left\{\phi_{1}^{j},\ldots ,\phi_{m}^{j}\right\}$ to be the new basis system for $H^{j}$. Then, we have

$$\begin{array}{c} {\left[X_{i}^{j}\right]}_{C^{j}}={\left(\Phi^{j}\right)}^{⊤}G^{j}{\left[X_{i}^{j}\right]}_{B^{j}},\;i=1,\ldots ,n,j=1,\ldots ,p. \end{array}$$

We assume that the coordinate of H is based on the orthonormal basis system C and drop the subscript for convenience throughout this section from this point. Then now we have,

$$\begin{array}{c} \left[{\hat{\Gamma }}_{XX}\right]=\text{diag}\left(\lambda_{1}^{1},\ldots ,\lambda_{m}^{1},\ldots ,\lambda_{1}^{p},\ldots ,\lambda_{m}^{p}\right),\;\left[X_{1:n}\right]=\text{diag}\left(\Phi_{1}^{⊤},\ldots ,\Phi_{p}^{⊤}\right)G{\left[X_{1:n}\right]}_{B}, \end{array}$$

and ${\langle f,g\rangle }_{H}={\left[f\right]}^{⊤}\left[g\right]$ for any f,g∈H.

### 4.3 Estimation

Using the representation, we can express the optimization (8) as follows.

$$\begin{array}{c} \begin{array}{c} {}[\beta^{\text{new}}]≔\text{arg}\;\underset{\beta \in H}{\text{min}}(f\left(\beta \right)+\frac{\rho }{2}{\left(\left[\beta \right]-\left[\gamma \right]+\left[U\right]\right)}^{⊤}\left(\left[\beta \right]-\left[\gamma \right]+\left[U\right]\right))\\ {}[\gamma^{\text{new}}]≔\text{arg}\;\underset{\gamma \in H}{\text{min}}(g\left(\gamma \right)+\frac{\rho }{2}{\left(\left[\beta^{\text{new}}\right]-\left[\gamma \right]+\left[U\right]\right)}^{⊤}\left(\left[\beta^{\text{new}}\right]-\left[\gamma \right]+\left[U\right]\right))\\ {}[U^{\text{new}}]≔\left[U\right]+\left[\beta^{\text{new}}\right]-\left[\gamma^{\text{new}}\right], \end{array} \end{array}$$ (9)

where

$$\begin{array}{cc} f(\beta ) & =\frac{1}{2}E_{n}{\left(Y-{\left\langle X,\beta \right\rangle }_{H}\right)}^{2}={\left(2n\right)}^{-1}\sum_{i=1}^{n}\left\{Y_{i}^{2}-2\left(Y_{i}⊗X_{i}\right)\beta +{\left\langle \beta ,\left(X_{i}⊗X_{i}\right)\beta \right\rangle }_{H}\right\}\\ & =\frac{1}{2}{\hat{\sigma }}_{YY}-{\hat{\Gamma }}_{YX}\beta +\frac{1}{2}{\left\langle \beta ,{\hat{\Gamma }}_{XX}\beta \right\rangle }_{H}=\frac{1}{2}{\hat{\sigma }}_{YY}-\left[{\hat{\Gamma }}_{YX}\right]\left[\beta \right]+\frac{1}{2}{\left[\beta \right]}^{⊤}\left[{\hat{\Gamma }}_{XX}\right]\left[\beta \right], \end{array}$$

and $g\left(\gamma \right)=\lambda \sum_{j=1}^{p}{\left\|\gamma^{j}\right\|}_{H^{j}}=\lambda \sum_{j=1}^{p}\sqrt{{\left[\gamma^{j}\right]}^{⊤}\left[\gamma^{j}\right]}$.

Under the finite-dimensional representation of the functional elements in H, one can see that the optimization in (9) is a convex optimization problem.

**Theorem 1** *The solution to the optimization problem* (3) *can be achieved by iterating over the following update rules*.

$$\begin{array}{ccc} & \left[\beta^{new}\right]={\left(\left[{\tilde{X}}_{1:n}\right]{\left[{\tilde{X}}_{1:n}\right]}^{⊤}+n\rho I_{M}\right)}^{-1}\left(\left[{\tilde{X}}_{1:n}\right]Y+n\rho \left(\left[\gamma \right]-\left[U\right]\right)\right) & \\ & \left[{\left(\gamma^{j}\right)}^{new}\right]=S_{\frac{\lambda }{\rho }}^{H^{j}}\left(\left[{\left(\beta^{j}\right)}^{new}\right]+\left[U^{j}\right]\right) & j=1\ldots p\\ & \left[U^{new}\right]=\left[U\right]+\left[\beta^{new}\right]-\left[\gamma^{new}\right], \end{array}$$ (10)

*where* [*γ*^*j*^], [*U*^*j*^] *are corresponding blocks to* [*β*^*j*^], *and* $S_{\lambda }^{H^{j}}\left(h\right)=1_{\left\{\|h{\|}_{H^{j}}>\lambda \right\}}(1-\frac{\lambda }{{\left\|h\right\|}_{H^{j}}}{)}_{+}h$ *for* $h\in H^{j}$.

If we do not consider orthogonalization, Theorem 1 would contain element *G*_*j*_ in the updates. In this case, the proof of numerical convergence of the update rules is slightly different from that of [23]. However, due to the orthogonalization, the proof of the numerical convergence of the updates in the Theorem 1 to the solution of the optimization problem (3) is identical to that of the ADMM in [23]. Hence, it is omitted.

### 4.4 Different penalty terms

In this section, we investigate the different penalty terms in two directions: one for the functional predictor selection, and the other one for the smooth coefficient functions *β*.

#### 4.4.1 Multivariate functional group Elastic net

LASSO does not provide a unique solution. To achieve uniqueness and overcome the saturation property, Elastic Net penalty has been introduced by combining the *ℓ*_1_-norm and *ℓ*_2_-norm by [12] for the multivariate data. Functional data are intrinsically infinite-dimensional objects. Thus, we propose a multivariate functional-version optimization problem for the Elastic net penalty by grouping each functional predictor as follows.

$$\begin{array}{c} \frac{1}{2}E_{n}{\left(Y-{\left\langle X,\beta \right\rangle }_{H}\right)}^{2}+\lambda \left(1-\alpha \right)\sum_{j=1}^{p}\|\beta^{j}{\|}_{H^{j}}+\alpha \lambda \sum_{j=1}^{p}{\left\|\beta^{j}\right\|}_{H^{j}}^{2}, \end{array}$$ (11)

where *α* ∈ [0, 1] and *λ* > 0 are the tuning parameters.

This optimization problem still follows the structure of the ADMM algorithm in (5) with $g\left(\gamma \right)=\lambda \left(1-\alpha \right)\sum_{j=1}^{p}\|\gamma^{j}{\|}_{H^{j}}+\alpha \lambda \sum_{j=1}^{p}{\left\|\gamma^{j}\right\|}_{H^{j}}^{2}$. It can be easily shown that the only difference from the original version is the *γ*-update in Theorem 1. Hence, we have the following update rules.

**Theorem 2** *The solution to the optimization problem* (11) *can be achieved by iterating over the following update rules*.

$$\begin{array}{ccc} & \left[\beta^{new}\right]={\left(\left[{\tilde{X}}_{1:n}\right]{\left[{\tilde{X}}_{1:n}\right]}^{⊤}+n\rho I_{M}\right)}^{-1}\left(\left[{\tilde{X}}_{1:n}\right]Y+n\rho \left(\left[\gamma \right]-\left[U\right]\right)\right) & \\ & \left[{\left(\gamma^{j}\right)}^{new}\right]=\frac{\rho }{\rho +2\alpha \lambda }S_{\frac{\lambda (1-\alpha )}{\rho }}^{H^{j}}\left(\left[{\left(\beta^{j}\right)}^{new}\right]+\left[U^{j}\right]\right) & j=1,\ldots ,p\\ & \left[U^{new}\right]=\left[U\right]+\left[\beta^{new}\right]-\left[\gamma^{new}\right]. \end{array}$$ (12)

Regularization parameters can be adjusted through a net search cross–validation.

#### 4.4.2 Smoothness of functional coefficients *β*

According to the simulation, we found that the previous algorithm provides a wiggly estimation of functional coefficients *β* most of the time. It might be fine if we are only interested in the prediction; however, it is not the case, because we consider the linear functional regression. We propose an algorithm that controls the roughness of *β* simultaneously to avoid the over-fitting problems and to obtain smooth functional coefficients. In particular, we impose the penalty on the curvature of the coefficients by adding $\frac{\lambda_{\text{der}}}{2}{\left\|\beta^{\prime \prime }\right\|}_{H}^{2}$ to the objective function (8). We include this term in *f*(⋅) function in the ADMM structure. Finally, the first update rule (10) in Theorem 1 becomes

$$\begin{array}{c} \left[\beta^{\text{new}}\right]:={\left(\left[{\tilde{X}}_{1:n}\right]{\left[{\tilde{X}}_{1:n}\right]}^{⊤}+n\rho I_{M}+\lambda_{\text{der}}G^{\prime \prime }\right)}^{-1}\left(\left[{\tilde{X}}_{1:n}\right]Y+n\rho \left(\left[\gamma \right]-\left[U\right]\right)\right), \end{array}$$ (13)

where *G*″ is a block-diagonal matrix whose *j*-th block matrix is ${\left({\left(G^{j}\right)}^{\prime \prime }\right)}_{ik}=\int_{T^{j}}{\left(\phi_{i}^{j}\right)}^{\prime \prime }\left(t\right){\left(\phi_{k}^{j}\right)}^{\prime \prime }\left(t\right)dt={\langle {\left(\phi_{i}^{j}\right)}^{\prime \prime },{\left(\phi_{k}^{j}\right)}^{\prime \prime }\rangle }_{H^{j}}$ for *i*, *k* = 1, …, *m*, *j* = 1 …, *p*.

For each *j*, (*G*^*j*^)″ can be derived from the second derivative Gram matrix for the original basis, say (*B*^*j*^)″, where ${\left({\left(B^{j}\right)}^{\prime \prime }\right)}_{ik}=\int_{T^{j}}{\left(b_{i}^{j}\right)}^{\prime \prime }\left(t\right){\left(b_{k}^{j}\right)}^{\prime \prime }\left(t\right)dt={\langle {\left(b_{i}^{j}\right)}^{\prime \prime },{\left(b_{k}^{j}\right)}^{\prime \prime }\rangle }_{H^{j}}$. Note that

$$\begin{array}{c} {\left[\phi_{i}^{j}\right]}_{B^{j}}={\left(G^{j}\right)}^{-1}{\left({\left(\Phi^{j}\right)}^{-1}\right)}^{⊤}{\left[\phi_{i}^{j}\right]}_{C^{j}}={\left(G^{j}\right)}^{-1}{\left({\left(\Phi^{j}\right)}^{-1}\right)}^{⊤}e_{i}, \end{array}$$

where *e*_*i*_ is *i*-th standard basis in $R^{m}$. Then,

$$\begin{array}{cc} {\left\langle {\left(\phi_{i}^{j}\right)}^{\prime \prime },{\left(\phi_{k}^{j}\right)}^{\prime \prime }\right\rangle }_{H^{j}} & ={\left\langle \sum_{\ell =1}^{m}{\left({\left[\phi_{i}^{j}\right]}_{B^{j}}\right)}_{\ell }{\left(b_{\ell }^{j}\right)}^{\prime \prime },\sum_{\ell =1}^{m}{\left({\left[\phi_{k}^{j}\right]}_{B^{j}}\right)}_{\ell }{\left(b_{\ell }^{j}\right)}^{\prime \prime }\right\rangle }_{H^{j}}\\ & =e_{i}^{⊤}{\left(\Phi^{j}\right)}^{-1}{\left(G^{j}\right)}^{-1}{\left(B^{j}\right)}^{\prime \prime }{\left({\left(G^{j}\right)}^{-1}{\left(\Phi^{j}\right)}^{-1}\right)}^{⊤}e_{j}. \end{array}$$

#### 4.4.3 Tuning

The initial values for *γ* and *U* are zero, and the initial *β* is the ridge regression estimation in the first update rule (10). We set the augmented parameter, a.k.a the step size, *ρ* to be 1 and stay the same through the algorithm. The different values of *ρ* only change the values of the optimal λ on the grid or optimal (1 − *α*)λ on the net. The larger the *ρ*, the smaller the optimized regularization parameter of the soft threshold operator. In some practices of augmented Lagrangian, it is possible to choose a small step size and increase it to 1 gradually in each iteration. It is also stated in [23] why *ρ* = 1 is a suitable choice in the ADMM algorithm.

We use the k-fold cross–validation for choosing the mixing parameter *α*, the regularization parameter of the second derivative penalty λ_der_, and the main regularization parameter λ. In particular, for each *α* and each λ_der_ on the net, we search for the optimal λ. To pick the initial λ, we first find the ridge estimation *β* with parameter *ρ* = 1. We then compute the norm of each of the groups of functional coefficients, ‖*β*^*k*^‖. Note that in the second update of Theorem 1, the soft threshold operator would eliminate all blocks if λ is slightly higher than the maximum of these norms. On the other hand, this update would keep all the coefficients if λ is slightly lower than the smallest norm. Therefore, a reasonable procedure is to design a grid of λ’s between a number slightly lower than the minimum norm of the blocks and a number slightly higher than the maximum norm of these block coefficients.

## 5 Estimation: GMD

In this section, we derive the GMD algorithm for solving the objective functions in Section 3. Unlike the ADMM, this algorithm is geared toward the objective function with group-wise penalty terms. Motivated by [26], we derive the GMD algorithm under our setting. In addition, we do not force the basis functions to be orthogonal, which allows us to have more flexibility. Thus, throughout this section, we use the coordinate system based on the original basis B without orthogonalization.

### 5.1 Algorithm

The MFG-Elastic Net problem without the orthogonalization is

$$\begin{array}{c} \text{arg}\;\underset{\beta }{\text{min}}\frac{1}{2}\|Y-{\left[{\tilde{X}}_{1:n}\right]}^{⊤}G\left[\beta \right]{\|}_{2}^{2}+\frac{\lambda_{\text{der}}}{2}{\left[\beta^{\prime \prime }\right]}^{T}G\left[\beta^{\prime \prime }\right]+\lambda \left(1-\alpha \right)\sum_{j=1}^{p}\|\beta^{j}{\|}_{H^{j}}+\alpha \lambda \sum_{j=1}^{p}{\left\|\beta^{j}\right\|}_{H^{j}}^{2}, \end{array}$$ (14)

where the coordinates are associated with the original basis B. This optimization problem and the following derived algorithm include the steps that also solve for the MFG-Lasso (*α* = 0) and the ridge regression (*α* = 1). In Eq (14), we remove *n* for computational convenience. It will be adjusted when we seek the λ_der_ and λ in the grid construction. We define the loss function as follows.

$$\begin{array}{c} L\left(\beta \right)=\frac{1}{2}{\left\|Y-{\left[{\tilde{X}}_{1:n}\right]}^{⊤}G\left[\beta \right]\right\|}_{2}^{2}+\frac{\lambda_{\text{der}}}{2}{\left[\beta^{\prime \prime }\right]}^{T}G\left[\beta^{\prime \prime }\right]. \end{array}$$ (15)

Consequently, the objective function (14) is *L*(*β*) + *g*(*β*) where $g\left(\beta \right)=\lambda \left(1-\alpha \right)\sum_{j=1}^{p}\sqrt{{\left[\beta^{j}\right]}^{⊤}G^{j}\left[\beta^{j}\right]}+\alpha \lambda \sum_{j=1}^{p}{\left[\beta^{j}\right]}^{⊤}G^{j}\left[\beta^{j}\right]$.

**Lemma 3** *The loss function* (15) *satisfies the quadratic majorization (QM) condition with* $H=G{\left[{\tilde{X}}_{1:n}\right]}^{⊤}\left[{\tilde{X}}_{1:n}\right]G+\lambda_{\text{der}}B^{\prime \prime }$. *In other words, for any* $\beta ,\beta^{*}\in H$,

$$\begin{array}{c} L\left(\beta \right)\leq L\left(\beta^{*}\right)+\left(\left[\beta \right]-\left[\beta^{*}\right]\right)\nabla L\left(\beta^{*}\right)+\frac{1}{2}{\left(\left[\beta \right]-\left[\beta^{*}\right]\right)}^{⊤}H\left(\left[\beta \right]-\left[\beta^{*}\right]\right), \end{array}$$ (16)

*where*,

$$\begin{array}{c} \nabla L\left(\beta^{*}|D\right)=G\left[{\tilde{X}}_{1:n}\right]\left({\left[{\tilde{X}}_{1:n}\right]}^{⊤}G\left[\beta \right]-Y\right)+\lambda_{\text{der}}B^{\prime \prime }\left[\beta^{*}\right], \end{array}$$ (17)

*where* |*D refers to condition given data, or given the design matrix*.

Let *U* = −∇*L*(β*). In addition to Lemma 3, it is straightforward to see that if *β* ≠ β*, we have the strict inequality,

$$\begin{array}{c} L\left(\beta |D\right)<L\left(\beta^{*}|D\right)-{\left(\left[\beta \right]-\left[\beta^{*}\right]\right)}^{T}U\left(\beta^{*}\right)+\frac{1}{2}{\left(\left[\beta \right]-\left[\beta^{*}\right]\right)}^{⊤}H\left(\left[\beta \right]-\left[\beta^{*}\right]\right). \end{array}$$ (18)

Thus, it leads to the strict descent property of the updating algorithm. Let β* be the current solution to the optimization problem and *β* be the next update. Assume that we update the *β* for each functional predictor *j* = 1, …, *p*. In other words, [*β*] − [β*] has a form of (0, …, 0, [*β*^*j*^] − [(β*)^*j*^], 0, …, 0)^⊤^, which leads to simplification of the objective function in the new optimization problem. Let *U*^*j*^ be the sub-vector of *U* with the indices (*m*(*j* − 1) + 1, …, *mj*). Let *H*^*j*^ be the *j*-th block diagonal matrix of *H*. Then, (16) is

$$\begin{array}{cc} L(\beta ) & \leq L\left(\beta^{*}\right)-\left(\left[\beta^{j}\right]-\left[{\left(\beta^{*}\right)}^{j}\right]\right)U^{j}+\frac{1}{2}{\left(\left[\beta^{j}\right]-\left[{\left(\beta^{*}\right)}^{j}\right]\right)}^{⊤}H^{j}\left(\left[\beta^{j}\right]-\left[{\left(\beta^{*}\right)}^{j}\right]\right)\\ & \leq L\left(\beta^{*}\right)-\left(\left[\beta^{j}\right]-\left[{\left(\beta^{*}\right)}^{j}\right]\right)U^{j}+\frac{1}{2}\gamma_{j}{\left(\left[\beta^{j}\right]-\left[{\left(\beta^{*}\right)}^{j}\right]\right)}^{⊤}\left(\left[\beta^{j}\right]-\left[{\left(\beta^{*}\right)}^{j}\right]\right), \end{array}$$

where *γ*_*j*_ is a value slightly larger than the largest eigenvalue of *H*^*j*^, which further relaxes the upper bound. In practice, we take *γ*_*j*_ = (1 + ϵ*)*η*_*j*_ with ϵ* = 10^−6^ where *η*_*j*_ is the largest eigenvalue of *H*^*j*^. Finally, the update rule for *β*^*j*^ is the solution to the following optimization problem.

$$\begin{array}{c} \text{arg}\;\underset{\beta^{j}\in H^{j}}{\text{min}}-\left(\left[\beta^{j}\right]-\left[{\left(\beta^{*}\right)}^{j}\right]\right)U^{j}+\frac{1}{2}\gamma_{j}{\left(\left[\beta^{j}\right]-\left[{\left(\beta^{*}\right)}^{j}\right]\right)}^{⊤}\left(\left[\beta^{j}\right]-\left[{\left(\beta^{*}\right)}^{j}\right]\right)+g^{j}\left(\beta \right), \end{array}$$ (19)

where *g*^*j*^ is the *j*-th term of *g*(⋅). We have a closed-form solution to this problem using a similar trick of Lemma 6 in the supplementary materials.

$$\begin{array}{c} {\left[\beta^{j}\right]}^{\text{(new)}}=\frac{1}{2\alpha \lambda +\gamma_{j}}S_{\lambda (1-\alpha )}^{H^{j}}\left(U^{j}+\gamma_{j}{\left[\beta^{j}\right]}^{\left(\text{old}\right)}\right),\;j=1,\ldots ,p, \end{array}$$ (20)

where *U*^*j*^ = −∇*L*(*β*^*j*(old)^) and $\nabla L\left(\beta \right)=G\left[{\tilde{X}}_{1:n}\right]\left({\left[{\tilde{X}}_{1:n}\right]}^{⊤}G\left[\beta \right]-Y\right)+\lambda_{\text{der}}B^{\prime \prime }\left[\beta \right]$.

### 5.2 Tuning parameter selection

While iterating over this GMD update rule, we can reduce the computational burden more efficiently during the tuning parameter selection with the strong rule technique. See [27].

*Step 1. (Initialization)* Given *α* ∈ (0, 1), the largest λ in the grid points is the smallest value of λ such that all its associated coefficients are zero. In particular, using the KKT condition (see Lemma 6 in the supplementary materials), the largest λ in the grid points is

$$\begin{array}{c} \lambda^{\left(1\right)}=\underset{j}{\text{max}}\frac{\|U^{j}\left(0\right)\|}{1-\alpha }. \end{array}$$

Therefore, the initial *β* is zero. Then, the smallest λ of the grid points is set to be a certain small number to include all the functional predictors, usually a fraction of the largest λ value of the grid. The process of searching for the optimal λ starts with the largest value of the grid points and moves backward to the smallest value.

*Step 2. (Iteration)* At λ^(*k*)^, we add *j*-th functional predictor to the active set if it satisfies the strong rule condition,

$$\begin{array}{c} \|U^{j}\left(\left[\beta^{j}\left(\lambda^{\left(k\right)}\right)\right]\right)\|>\left(2\lambda^{\left(k+1\right)}-\lambda^{\left(k\right)}\right)\left(1-\alpha \right), \end{array}$$

for *j* = 1, …, *p*. Subsequently, we update *β* with these reduced predictors by iterating the update rule (20) until numerical convergence. The stopping criteria for this iterative process can be chosen the absolute or relative. Next, to make sure that the strong rule does not leave out some of the worthy coefficients, we check the KKT condition on the rest of the blocks of the current solution,

$$\begin{array}{c} \|U^{j}\left(\left[\beta_{update}^{j}\left(\lambda^{\left(k+1\right)}\right)\right]\right)\|<\lambda^{\left(k+1\right)}\left(1-\alpha \right), \end{array}$$

where $\beta_{update}^{j}\left(\lambda^{\left(k+1\right)}\right)$ is the updated *β*^*j*^ when the iterative GMD algorithm hits the stopping criteria on the result of the strong rule screening. If *j*-th functional coefficient violates the KKT condition, we add it to the active set and update *β* using (20). This process of checking the KKT condition and updating, continues until there is no functional coefficient that violates the KKT condition. We store the solution of the final updated value to *β*^*j*^(λ^(*k*+1)^). We use *β*^*j*^(λ^(*k*+1)^) to repeat *(Step 2)* for the next value of λ (warm start).

It is worth mentioning that the strong rule does not allow that the main regularization for λ to be computed in parallel because of the warm start, i.e. we search for λ sequentially. However, the main computational cost is paid in this regularization. The strong rule allows the algorithm to enjoy predictor screening, which leads to a cost-effective computation by storing and computing on smaller size vectors. On the other hand, the strong rule does not seem to be valid for the ADMM algorithm because there are two objective functions involved in this algorithm. Hence, it is possible to tune the regularization parameters in parallel via ADMM.

## 6 Asymptotic results

In this section, we derive the consistency of the multivariate functional group LASSO (MFG-LASSO) when functions are fully observed. It is worth mentioning that the regularization parameter of the second derivative penalty term in the loss function is zero when the number of time points and the number of basis are infinity, i.e. fully observable. Therefore, the asymptotic properties of such a model are considered without the curvature penalty when we assume fully observable functional covariates. In particular, the consistency breaks down to the selection consistency and the estimation consistency, which is known as the oracle property.

We first illustrate the consistency of the operators used in the estimation procedure. Since the implementation in Section 4.1 is based on the method of moments estimate, the following lemma is an immediate result from the functional-version of the central limit theorem in a separable Hilbert space. See [22].

**Lemma 4** *If* ${E\|X\|}_{H}^{4}<\infty$ *and EY*^4^ < ∞, *then*

1.
$\sqrt{n}({\hat{\Gamma }}_{XX}-\Gamma_{XX})\overset{D}{\to }N(0,\Sigma_{XX})$ ,
2.
$\sqrt{n}({\hat{\Gamma }}_{YX}-\Gamma_{YX})\overset{D}{\to }N(0,\Sigma_{YX})$ ,
3.
$\sqrt{n}({\hat{\Gamma }}_{YY}-\Gamma_{YY})\overset{D}{\to }N(0,\Sigma_{YY})$ ,

*where* Σ_*XX*_ = *E*[{(*X* − *EX*) ⊗ (*X* − *EX*) − Γ_*XX*_} ⊗ {(*X* − *EX*) ⊗ (*X* − *EX*) − Γ_*XX*_}] *and* Σ_*YX*_, Σ_*YY*_ *are similarly defined*.

Now, we limit our index to *J*, the true active set of the population functional coefficient *β*. For convenience, we use the notation for truncated-version by the superscript *J* such that $\beta^{J}=\left(\beta^{j}:j\in J\right)\in H^{J}$.

**Lemma 5** *In addition to the assumptions in Lemma 4, assume that for any j, there exists g*^*j*^ ∈ *H*^*j*^ *such that* $\beta^{j}=\Gamma_{X^{j}X^{j}}^{1/2}\left(g^{j}\right)$. *This means each β*^*j*^ *is in the range of* $\Gamma_{X^{j}X^{j}}^{1/2}$. *Consider* $\beta_{n}^{J}$ *as a minimizer of*

$$\begin{array}{c} \frac{1}{2}E_{n}\left[{\left(Y-{\left\langle X^{J},\beta^{J}\right\rangle }_{}\right)}^{2}\right]+\lambda_{n}\sum_{j\in J}{\left\|\beta^{j}\right\|}_{H^{j}}. \end{array}$$ (21)

*If* λ_*n*_ → 0 *and* $\lambda_{n}\sqrt{n}\overset{}{\to }\infty$, *then* $\|\beta_{n}^{J}-\beta^{J}{\|}_{H}$ *converges to zero in probability, slightly slower than* $\sqrt{\lambda_{n}}+\lambda_{n}^{-1}n^{-1/2}$.

The above lemma illustrates that if we know the true functional predictors, the solution to the optimization problem (3) achieves consistency. Let *M*_*n*_(⋅) be the objective function in (21). Then,

$$\begin{array}{c} M_{n}\left(\beta \right)=\frac{1}{2}{\hat{\Gamma }}_{YY}-{\hat{\Gamma }}_{YX^{J}}\beta +\frac{1}{2}{\left\langle \beta ,{\hat{\Gamma }}_{X^{J}X^{J}}\beta \right\rangle }_{}+\lambda_{n}\sum_{j\in J}{\left\|\beta^{j}\right\|}_{H}. \end{array}$$ (22)

Note that (22) is asymptotically strictly convex as long as we can assume that $\Gamma_{X^{J}X^{J}}$ is a positive-definite operator. Similarly, the original objective function (3) has also a unique solution if we can assume that Γ_*XX*_ exists and is positive definite. By using Lemma 5 as a bridge, we prove the consistency of our estimate in the following theorem.

**Theorem 3** *Assume that*

1. *The fourth moments of X and Y are bounded*.
2. *For any j, there exists g*^*j*^ ∈ *H*^*j*^ *such that* $\beta^{j}=\Gamma_{X^{j}X^{j}}^{1/2}\left(g^{j}\right)$.
3. *In the population, we have such a condition that*,
  $$\begin{array}{c} \underset{i\in J^{c}}{\text{max}}\|\Gamma_{X^{i}X^{i}}^{1/2}C_{X^{i}X^{J}}C_{X^{J}X^{J}}^{-1}diag(\left(\cdot \right)/\|\beta^{j}{\|}_{H}{)\left(g^{J}\right)\|}_{H^{J}}<1, \end{array}$$
  *where* $C_{X^{i}X^{J}}$ *and* $C_{X^{J}X^{J}}$ *are the correlation operators defined in* [28]- $\Gamma_{X^{i}X^{J}}=\Gamma_{X^{i}X^{i}}^{1/2}C_{X^{i}X^{J}}\Gamma_{X^{J}X^{J}}^{1/2}$.

*Then, the multivariate functional group LASSO estimate satisfies the following*.

1. *Let* $\hat{\beta }$ *be the solution minimizing* (3), *and* $\hat{J}=\left\{j;{\hat{\beta }}^{j}\neq 0\right\}$ *be the estimated active set*. *Then*, $P(\hat{J}=J)$ *converges to 1*.
2.
$\|\hat{\beta }{-\beta \|}_{H}\to 0$ *in probability if* λ_*n*_ *approaches zero slower than the rate of n*^−1/2^.

Assumption 1 is commonly used in the conditions of the functional central limit theorem. In addition, such an assumption guarantees the decay of the eigenvalues of the covariance operator of *X*. Assumption 2 states that the functional coefficients *β*^*j*^ lies in the support of the functional predictor *X*, which means that we restrict the potential range of *β* to be in the range of Σ_*XX*_. Assumption 3 is a modified version of the necessary condition for the LASSO to be consistent that is derived in [14]. In fact, this assumption states intuitively that the correlation between a truly inactive covariate and all truly active covariates is bounded by an upper bound, so the active covariates do not drag or pull the indices of non-active covariates in the final active set when the sample size grows. This assumption will be used in the proof of selection consistency.

The rate of convergence is at most $O_{p}\left(\sqrt{\lambda_{n}}+n^{-1/2}\lambda_{n}^{-1}\right)$. This is the upper bound of the rate of estimation convergence in Lemma 5 when the true active set is known and indices are limited to it.

It is worth mentioning that the natural rivals of the proposed models such as group sparse regression models (group LASSO and group Elastic Net) without basis transformation do not provide a smooth estimation. In addition, they are extremely slow to estimate due to a large number of time points in the data; hence, in the following two sections (simulation and application), we do not include them for comparison with the proposed methods.

## 7 Simulation studies

In this section, we investigate the performance of the proposed method for scalar on functional penalized regressions through a simulation study when the set of time points are the same for all the observed functional data. We also study the unbalanced version but the behavior of the performance is similar to the balanced case except that the unbalanced one is slightly worse. Thus, we summarize the results from the unbalanced case in the supplementary material.

Consider *T* = [0, 1] with a hundred observed time points equally-spaced, {*t*_1_, …, *t*_100_}. Suppose that there are *p* = 19 random functional covariates, *X*^*j*^, for *j* = 1, …, 19, observed on a hundred time points equally-spaced in *T* = [0, 1], say {*t*_1_, …, *t*_100_}. For *i* = 1, …, *n*, we first generate $X_{i}=\left(X_{i}^{1},\ldots ,X_{i}^{p}\right)$ on 500 time points, $\left\{t_{1}^{*},\ldots ,t_{500}^{*}\right\}$, where $X_{i}^{j}$ is from a form of the Brownian motion,

$$\begin{array}{c} X^{j}\left(t_{i}^{*}\right)=\sum_{k=1}^{i}N_{k}^{j}, \end{array}$$

where 1 ≤ *k* ≤ 500, $N_{k}^{j}∼N\left(0,1\right)$. We generate the response values following the model

$$\begin{array}{c} Y={\left\langle X^{1},\beta^{1}\right\rangle }_{}+{\left\langle X^{2},\beta^{2}\right\rangle }_{}+{\left\langle X^{3},\beta^{3}\right\rangle }_{}+\sigma \epsilon , \end{array}$$

where *ϵ* ∼ *N*(0, 1), $\beta^{1}\left(t\right)=\text{sin}\left(\frac{3\pi t}{2}\right)$, $\beta^{2}\left(t\right)=\text{sin}\left(\frac{5\pi t}{2}\right)$, and *β*^3^(*t*) = *t*^2^ that are elements of $H^{j}$ for *j* = 1, 2, 3. Therefore, there are three functional predictors out of 19 in the population active set, *J* = {1, 2, 3}. We drop 400 observed time points so that the remaining 100 time points are equally spaced over [0, 1]. To compute the inner product with more accuracy, we used 500 points in Riemann sum approximation of the inner product integrals before dropping the 400 time points.

To investigate the method thoroughly, we applied different numbers of observations (100, 200, 500) and different standard deviations for the residual term *σ* = 0.01, 0.1, 1. In each sample, we divide the observations into two sets for training and test sets (80% for the training set, and 20% for the test set). We measure the root mean squared error (RMSE) of the prediction for the response values of the test set. In addition, we measure the number of functional predictors that are chosen correctly. More specifically, we count the correctly identified functional predictors in the population active set, the size of which is 3, and in the population inactive set, the size of which is 16 while predicting the test response values. With a cross-validation on the number of basis between 5 and 110, and the prediction error criteria, we choose *m* = 21 B-spline basis functions to convert the observed values to functional objects and coordinate representations. The second derivative penalty would guarantee that we do not overfit the curve estimations -after some number of basis, the curve estimations remain the same. We use 5-fold cross–validation to tune the regularization parameters on a net.

In each scenario, we generate 100 samples and compute the percentages of correctly selected functional predictors that are tabulated in Table 1, and compute the mean and standard deviation of the test RMSE that are in Table 2. Furthermore, we compare the sparse methods along with the scalar on functional ordinary least square method (OLS), ridge regression, and the oracle procedure in which only the functional predictors in the population active set are used in the OLS. For the sparse models, we apply the multivariate functional group LASSO (MFG-LASSO), and the MFG-Elastic Net (MFG-EN). The two algorithms, GMD with the strong rule and ADMM, provide similar results while the GMD algorithm is much faster on serial systems and ADMM is faster on parallel computational systems. Thus, we show the results using the GMD and strong rule algorithm in this paper.

**Table 1:** Percentages of correct selection in the test set under various simulation scenarios. In each case, 100 random samples are used. In each sample, we count the correctly identified functional predictors for the active set of the size 3 and the inactive set of the size 16. Then, we compute the overall percentage out of 100 samples.

Parameters		Selection	Methods			
*σ*	*n*		OLS	Ridge	MFG-LASSO	MFG-EN
0.01	100	Inactive	0	0	76	66
		Active	100	100	100	100
	200	Inactive	0	0	93	88
		Active	100	100	100	100
	500	Inactive	0	0	100	99
		Active	100	100	100	100
0.1	100	Inactive	0	0	73	64
		Active	100	100	100	100
	200	Inactive	0	0	92	86
		Active	100	100	100	100
	500	Inactive	0	0	100	99
		Active	100	100	100	100
1	100	Inactive	0	0	25	21
		Active	100	100	100	100
	200	Inactive	0	0	29	24
		Active	100	100	100	100
	500	Inactive	0	0	51	44
		Active	100	100	100	100

**Table 2:** Average test RMSE of different methods under different simulation scenarios. In each case, 100 random samples are used to compute the mean and standard deviation with parentheses.

Parameters		Methods				
*σ*	*n*	OLS	Ridge	MFG-LASSO	MFG-EN	Oracle
0.01	100	1.57	2.41	1.01	1.02	0.9
		(0.47)	(0.54)	(0.55)	(0.55)	(0.61)
	200	0.7	1.22	0.75	0.76	0.66
		(0.45)	(0.35)	(0.43)	(0.43)	(0.47)
	500	0.48	0.72	0.56	0.57	0.47
		(0.3)	(0.22)	(0.26)	(0.26)	(0.31)
0.1	100	1.6	2.41	1.02	1.03	0.91
		(0.47)	(0.55)	(0.55)	(0.54)	(0.6)
	200	0.73	1.22	0.76	0.77	0.67
		(0.44)	(0.35)	(0.43)	(0.42)	(0.47)
	500	0.5	0.73	0.58	0.58	0.49
		(0.29)	(0.22)	(0.26)	(0.26)	(0.3)
1	100	2.99	2.82	1.64	1.67	1.5
		(0.65)	(0.57)	(0.44)	(0.45)	(0.44)
	200	1.95	1.8	1.37	1.38	1.32
		(0.32)	(0.31)	(0.31)	(0.31)	(0.31)
	500	1.38	1.37	1.21	1.21	1.18
		(0.19)	(0.17)	(0.17)	(0.17)	(0.18)

From Table 1, we can see that the MFG-sparse methods effectively select the correct functional predictors. It also shows consistency in an empirical way. In particular, they always select the active set correctly even with a large noise, but the selection performances of eliminating the inactive set predictors are poor with a small sample or large noise. The MFG-EN tends to choose more functional predictors than others. It is an expected result since the MFG-EN penalty includes the quadratic term which gives more stability but tends to choose more predictors. Because the Oracle estimator assumes that the truly active and inactive sets are known before OLS is run on the sample with the indices of the true active set, it always hits 100 percent when selecting the active and the inactive indices in this table; hence, we do not display the results of this estimator in this table.

Table 2 illustrates the estimation performance using the test RMSE. The overall behavior of the methods in terms of prediction errors is similar to that of their selection performance. As the sample size grows, the RMSEs are closer to that of the oracle estimator and their standard deviations decrease. Compared to the OLS, the sparse methods outperform when there are not enough observations or the functions are noisy. The OLS performs slightly better than the sparse methods when we have large enough *n* and small noises. However, the standard errors of the OLS RMSE are larger than that of the MFG-methods. The ridge method is worse than the OLS with the small noise, but it is better than the OLS with the large noise. Overall, the sparse methods, MFG-LASSO and MFG-EN, perform the best in general because their results are very close to the oracle estimations. Considering that the sparse methods use much fewer functional predictors, the simulation results illustrate the great effectiveness of our methods in reducing both the model complexity and the prediction error.

**Fig 2:**
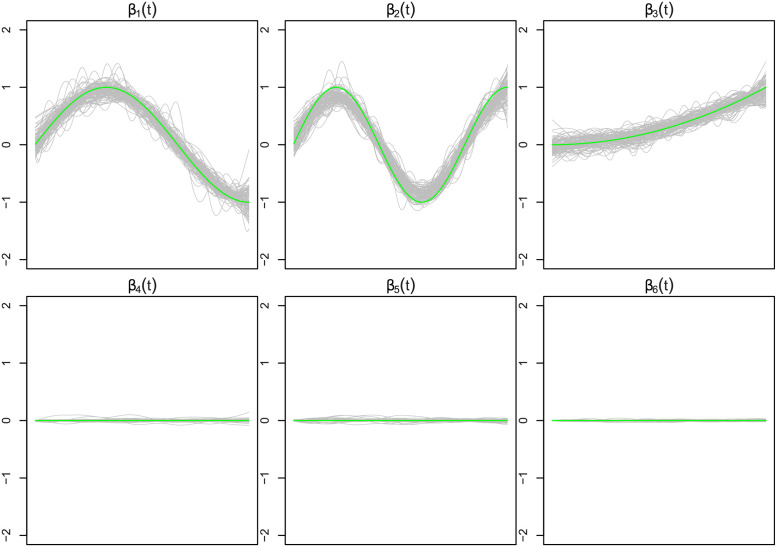
This figure displays the estimated functional coefficients by the MFG-LASSO from a hundred simulated data sets when *n* = 100, *σ* = 1. The green curves are the true coefficient curves and the grey curves are the estimated coefficients. The estimated curves for the remaining of the coefficients from the seventh to the nineteenth are very similar to the fourth, fifth, and sixth functions (inactive coefficients) displayed in this figure.

**Fig 3:**
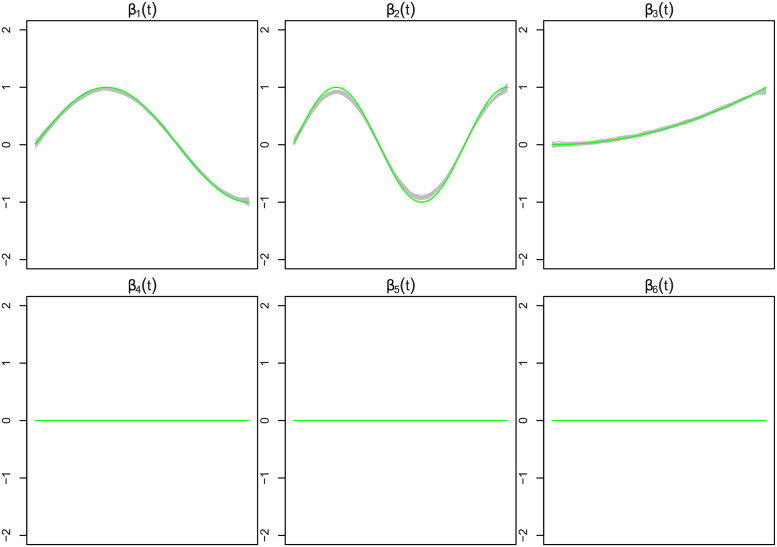
This figure displays the estimated functional coefficients by the MFG-LASSO from a hundred simulated data sets when *n* = 500, *σ* = 0.01. The green curves are the true coefficient curves and the grey curves are the estimated coefficients. The estimated curves for the remaining of the coefficients from the seventh to the nineteenth are very similar to the fourth, fifth, and sixth functions (inactive coefficients) displayed in this figure.

Fig 2 shows the estimated functional coefficients ${\hat{\beta }}^{1}\left(\cdot \right),\ldots ,{\hat{\beta }}^{6}\left(\cdot \right)$ from the MFG-LASSO in a hundred simulation samples when *n* = 100, *σ* = 1, the worst performance case. It must be mentioned that the estimations are individually smooth (for each of the 100 simulations) as they should be because of the curvature penalty. However, the estimated curves for 100 samples are highly variant due to the large noise. Thus, the curves do not look smooth when they are displayed in a single figure. The green curves are the true functions, and the rest of the curves are the estimations. Fig 3 shows the results when *n* = 500, *σ* = 0.01, the best performance case.

## 8 Applications to fMRI

We apply our methods to a human brain fMRI data set collected by the New York University. This data set is part of the ADHD-200 resting-state fMRI and anatomical datasets. The parent project is 1000 Functional Connectomes Project. The BOLD-contrast activities of the brain are measured by the fMRI machine during a 430 seconds period of time. To extract the time courses, 172 equally-spaced signal values were recorded as the observed points within the 430 seconds period of time. Before the analysis, the automated anatomical labeling (AAL) [29] was applied to the raw fMRI data by averaging the BOLD activities of the clusters of voxels in *p* = 116 regions of the brain, the regions of interest (ROI). This procedure is called masking, clustering the voxels by regions and averaging the time series signals within the region. The data consists of between five to seven brain resting–state fMRI records taken from 290 human subjects. We randomly choose two brain images from each human subject and clean the data by removing missing response values. We choose different response values in each regression analysis, such as the subjects’ intelligence quotient (IQ) scores, verbal IQ, performance IQ, attention deficit hyperactivity disorder (ADHD) index, ADHD Inattentive, and ADHD Hyper/Impulsive. Then, we split the data by 80% for the training set and 20% for the test set. Using cross-validation on the number of basis between 10 and 110, and the prediction error criteria, we choose *m* = 31 Fourier basis functions in the function approximation procedure.

Table 3 describes the test RMSE and the sparsity of the regression models. The results show that the scalar on function OLS does not work in that the RMSE is higher than the standard deviation of the response values in the test set. The ridge regression has a significantly lower RMSE while it does not select functional covariates. The MFG-LASSO eliminates more than half of the brain regions except for the performance IQ, while its RMSE is slightly higher than the MFG-EN in most cases. In terms of the RMSE, the MFG-EN performs the best while it selects more functional predictors than the MFG-LASSO. It is worth mentioning that when we change the proportion of the train and test data set to 90% and 10%, the ratio $\frac{RMSE}{{\hat{\sigma }}_{{\text{Y}}_{\text{test}}}}$ decreases significantly for sparse regressions; however, to be consistent with the simulations, we keep the 80% to 20% proportions for the train and test sets.

**Table 3:** The results of applying the proposed methods to the fMRI data when predicting the IQ and ADHD scores.

Response value	Method	RMSE	Zero curves of 116 ROI
Y = IQ score	Least square	19.01	0
Range: 73 − 142	Ridge	5.98	0
${\hat{\sigma }}_{{\text{Y}}_{\text{test}}}=13.45$	MFG-LASSO	6.32	63
	MFG-EN	5.91	10
Y = Verbal IQ	Least square	23.03	0
Range: 65 − 143	Ridge	7.02	0
${\hat{\sigma }}_{{\text{Y}}_{\text{test}}}=13.25$	MFG-LASSO	6.98	68
	MFG-EN	6.44	16
Y = Performance IQ	Least square	19.69	0
Range: 72 − 137	Ridge	6.27	0
${\hat{\sigma }}_{{\text{Y}}_{\text{test}}}=13.89$	MFG-LASSO	6.79	40
	MFG-EN	6.06	10
Y = ADHD Index	Least square	28.86	0
Range: 40 − 99	Ridge	8.18	0
${\hat{\sigma }}_{{\text{Y}}_{\text{test}}}=15.22$	MFG-LASSO	8.49	75
	MFG-EN	8.06	25
Y = ADHD Inattentive	Least square	27.81	0
Range: 40 − 90	Ridge	8.40	0
${\hat{\sigma }}_{{\text{Y}}_{\text{test}}}=15.30$	MFG-LASSO	9.21	75
	MFG-EN	8.67	30
Y = ADHD Hyper/Impulsive	Least square	26.47	0
Range: 41 − 90	Ridge	7.66	0
${\hat{\sigma }}_{{\text{Y}}_{\text{test}}}=14.66$	MFG-LASSO	8.42	60
	MFG-EN	8.54	52

At the time of writing, no research study uses the same data. However, some articles predict the IQ score based on human brain measurements. [30] predicted IQ score based on structural magnetic resonance imaging (MRI). To predict the IQ score, they use two methods: Principal component analysis on gray matter volume of each voxel, and Atlas-based grey matter volume while adjusting for the brain size in both methods. The reported RMSE with 90% to 10% train to test proportions in this study is 13.07 at its best, while the standard deviation of the IQ scores in the whole sample including the test set is ${\hat{\sigma }}_{Y}=12.94$. Nevertheless, the MFG-LASSO provides an RMSE of 6.32 and the MFG-EN provides 5.91. In addition, to the higher accuracy, our methods have much less complexity of the model. [30] selects more than 20, 000 principal features among all of the features associated with 556, 694 voxels in the data. Meanwhile, our methods use 53 functional predictors for MFG-LASSO and 106 functional predictors for MFG-EN. In each functional predictor, we use 172 time points in the raw data. Therefore, the proposed methods have obvious advantages in reducing the model complexity as well as achieving higher accuracy. Running one regression analysis with the proposed methods using the GMD/Strong Rule is on average around two to three minutes on a dual Core-i7 CPU with 16 GB memory, while the mentioned article claims an equivalent computation of 36, 000 hours using two CPU kernels and 5 GB RAM. In addition, there is another research study, [31]. In this article, the RMSE does not get any better than around 14 while data is from a combination of resting–state and task fMRI, and the sparse method uses voxels’ functional connectivities (Pearson correlation between BOLD time series signals) as the input features.

**Fig 4:**
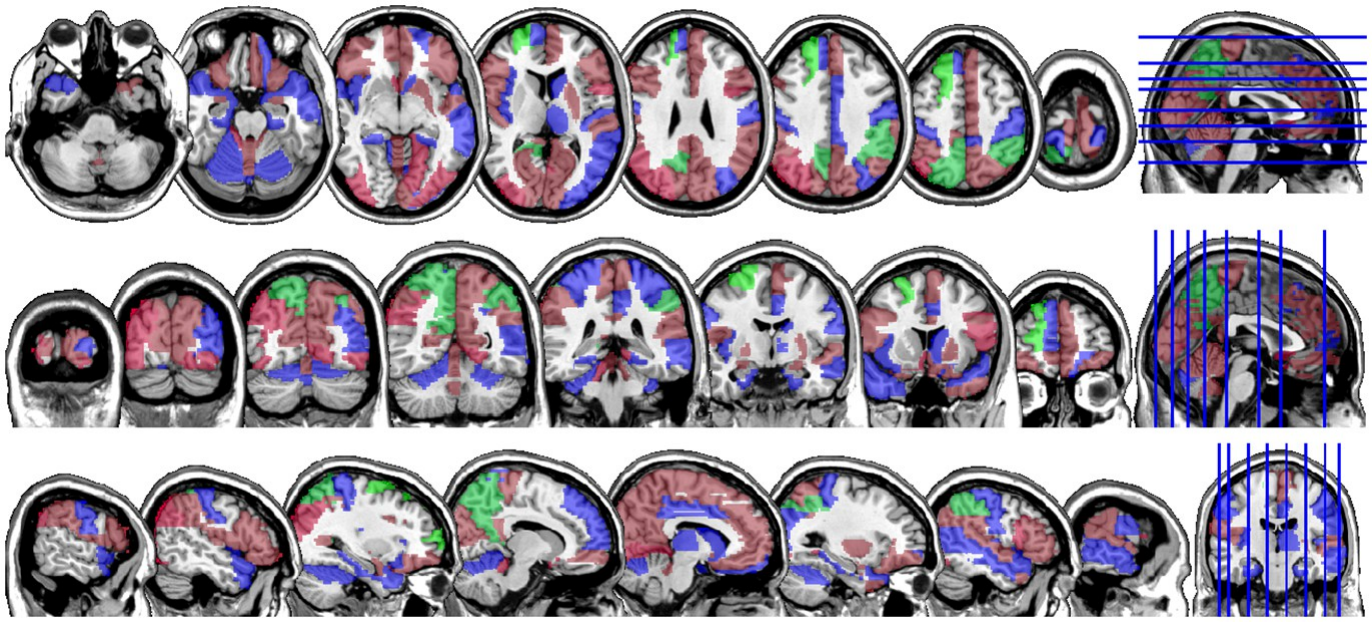
The multi-slice display (Axial, Coronal, Sagittal) of the regions of interests, the BOLD activities of which achieves the lowest prediction error and correlate the most with the IQ score variability in the sample when the MFG-LASSO is used. The regions associated with the IQ score are colored red, those associated with the performance IQ are blue, and the ones associated with the verbal IQ are colored green.

**Fig 5:**
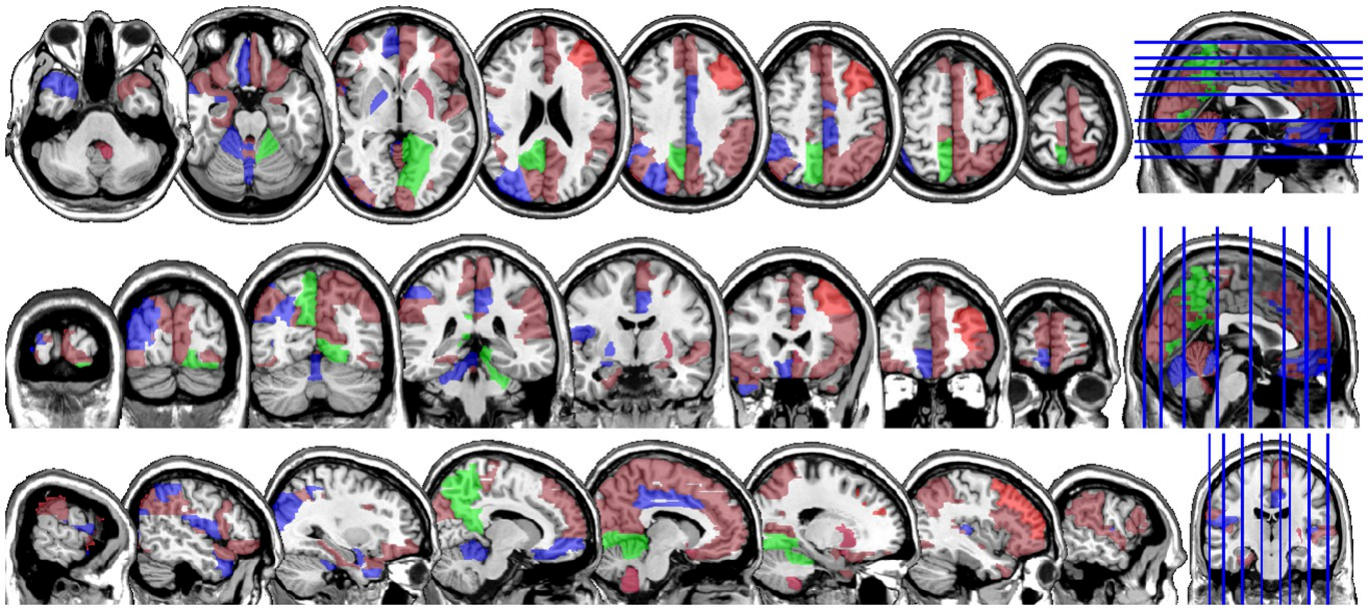
The multi-slice display (Axial, Coronal, Sagittal) of the regions of interests, the BOLD activities of which achieves the lowest prediction error and correlate the most with the ADHD score variability in the sample when the MFG-LASSO is used. The regions associated with the ADHD score are colored red, those associated with the ADHD Hyper/Impulsive are blue, and the ones associated with the ADHD Inattentive score are colored green.

In Figs 4 and 5, we display the regions associated with the estimated active sets for IQ and ADHD by the MFG-LASSO respectively. The final active sets of the algorithms were extracted and matched with the AAL’s atlas where each of the regions has a label. The regions were manually entered into the WFU picked atlas [32] tool of the SPM-12 ran on MATLAB 2020b to produce mask.nii files. The mask files were imported on MRIcron software to produce the multi-slice images.

The active sets cover the regions associated with IQ in [33] such as cerebello-parietal component and the frontal component. It is mentioned in the paper that the parietal and the frontal regions are strongly associated with intelligence by maintaining a connection with the cerebellum and the temporal regions. The shaded areas cover the ones mentioned in [34] as well. We provide the name of the regions associated with these active sets in the supplementary materials.

Interestingly, ADHD and IQ have a large proportion of common active sets. For instance, when MFG-LASSO is applied, they overlap in 35 ROIs where the size of active sets are 53 and 41 for IQ and ADHD respectively. On the other hand, the ROIs that are associated with ADHD but not with IQ are the middle and superior frontal, the Parahippocampal, the inferior parietal, and the superior temporal pole gyri. The ratio of the number of right hemisphere regions to the left ones associated with IQ is significantly greater than that of ADHD.

## 9 Conclusion

We propose new methods for scalar-on-function regression with the functional predictor selection along with the estimation of smooth coefficient functions when the predictors are multivariate functional data. We derive the algorithm for the implementation and develop the consistency of the methods by showing its oracle property. The simulation and real data application show the effectiveness of the methods with the superior performance of the proposed penalized methods over the functional regression model with the OLS. Furthermore, the proposed methods provide higher accuracy as well as the low complexity of the model in the fMRI study. It shows that there is an urgent need in the fields of medical sciences and other related areas.

The manuscript also has a potential impact on the field of statistical research for more advanced sparse functional models. Considering that there is not enough investigation made to sparse modeling of multivariate functional data, the computation algorithm derived in this paper will pave the way to develop other novel sparse methods. In addition, the methods can be extended to the nonlinear regression model via the reproducing kernel Hilbert space (RKHS). Since the theoretical justification is constructed under the infinite-dimensional setting, the extension on the RKHS can easily adopt the results from this paper. Furthermore, the proposed methods are based on groups such that a single functional predictor forms a group. Hence, it can be easily extended to the sparse models where multiple functional predictors form a group. For example, instead of averaging out fMRI signals of voxels over the regions of the brain, we would keep the original data and apply the MFG methods with groups formed by each region’s voxels activities. Then, we might figure out a new foundation that has been removed in the masking procedure.

In addition, extensions of the proposed methods can be applied to a wide range of research areas. Extending the result to binary response values can have applications in block design fMRI experiments where a stimulus status is on or off for all subjects at the same time. This model can then select ROIs or voxels associated with the stimulus. Furthermore, such an extension can be used to classify the ROI or voxels associated with a disease in a case–control study. Standardization of the results by estimating the standard deviation of the norm of the estimated coefficient curves can lead to a rank analysis of the ROI or voxels in the final active set of the sparse models. Such a rank analysis determines the importance of each ROI or voxel in the final active set and would reveal the curves that are weak signal and large noise. Aside from these two potential extensions and their fMRI applications, extension to functional response values can have an important application in event-related design task fMRI experiment data analysis where response values are a binary time series of a stimulus status that is randomly on or off for each subject in time.

## Supporting information

S1 File Supplementary materials. (PDF)

S1 Table Average test RMSE of different methods under different simulation scenarios when we have unbalanced time points for each observation. (PDF)

S2 Table Percentages of correct selection in the test set under various simulation scenarios when we have unbalanced time points for each observation. (PDF)
