# Correction: Multivariate functional group sparse regression: Functional predictor selection

**DOI:** 10.1371/journal.pone.0292772

**Published:** 2023-10-05

**Authors:** Ali Mahzarnia, Jun Song

There are errors in the Funding section. The correct Funding statement is: Jun Song is supported by a Korea University Grant (K2127931), URL:korea.ac.kr, and the National Research Foundation of Korea(NRF) grant funded by the Korea government(MSIT) (No. 2022R1C1C1003647), URL:nrf.re.kr. The funders had no role in study design, data collection and analysis, decision to publish, or preparation of the manuscript.
