# Supplementary material for: Multivariate functional group sparse regression: Functional predictor selection
Source: PLoS One. 2022 Apr 7;17(4):e0265940. doi: 10.1371/journal.pone.0265940 (PMC8989243; doi:10.1371/journal.pone.0265940)
Supplement: S1 File — (PDF) [file pone.0265940.s001.pdf]

# Supplementary materials of Multivariate functional group sparse regression: functional predictor selection

Ali Mahzarnia, Jun Song*

**1** Department of Mathematics and Statistics, University of North Carolina at Charlotte, Charlotte, NC 28223, USA
**2** Department of Statistic, Korea University, Seoul, South Korea

*junsong@korea.ac.kr

## 1 Simulation Results for Unbalanced observations

We investigate the performance further for the unbalanced observations. The sampling procedure is conducted in a similar way of the balanced version. In the balanced version, we generate the data set using equally-spaced 100 time points out of the data on 500 time points. In the unbalanced version, we choose 100 time points randomly for each observation, $i = 1, \ldots, n$. Then we generate 100 samples for each scenario and summarize the results in S1 Table and S2 Table.

The table in S1 Table shows the estimation consistency based on the RMSE for each case. We can see that our sparse methods outperform the OLS and the ridge penalty and the results are very close to the oracle. We can also see that the difference from the oracle becomes smaller as $n$ increases.

The table in S2 Table shows the performance of the selection performance. Even with the unbalanced case, the methods always choose the active functional predictors correctly as the balanced case, but the it tends not to remove the inactive functional predictors. The major reason for that is because of the model we used. We generate the functional data $X$ based on the random walk. It is obvious that the balanced observation has a strong advantage against the unbalanced case in the function estimation procedure. Overall, considering the estimation performance, even with this disadvantage, the functional group sparse methods work very well.

## 2 Proof

**Proof of Lemma 1** The representation of $[\hat{\Gamma}_{XX}]$ can be shown by the relation between the two following equations.

$$\langle f, \hat{\Gamma}_{XX} g\rangle_{\mathcal{H}} = E_n(\langle f, X - E_n X\rangle_{\mathcal{H}} \langle g, X - E_n X\rangle_{\mathcal{H}}) = [f]_{\mathcal{B}}^{\mathsf{T}} G [X_{1:n}]_{\mathcal{B}} Q [X_{1:n}]_{\mathcal{B}} [g]_{\mathcal{B}},$$
$$\langle f, \hat{\Gamma}_{XX} g\rangle_{\mathcal{H}} = [f]_{\mathcal{B}}^{\mathsf{T}} [\hat{\Gamma}_{XX}] [g]_{\mathcal{B}},$$

for any $f, g \in \mathcal{H}$. The second equation can be shown as following. For any $\beta \in \mathcal{H}$,

$$\begin{aligned}\hat{\Gamma}_{YX}\beta &= E_n\{(Y - E_n Y) \otimes (X - E_n X)\}\beta = E_n\{(Y - E_n Y)\langle X - E_n X, \beta\rangle_{\mathcal{H}}\} \\ &= E_n\{(Y - E_n Y)[X - E_n X]^{\mathsf{T}} G[\beta]\}.\end{aligned}$$

We can also see that $\hat{\Gamma}_{XY} = n^{-1}[\tilde{X}_{1:n} Y]$. □

**Lemma 6** *Take* $x, y \in \mathbb{R}^m$ *where* $y$ *is known.*

$$\arg\min_x \left(\frac{1}{2}\|x-y\|^2 + \lambda\|x\|\right) = S_\lambda(y), \qquad (23)$$

*where* $S_\lambda(y) := 1_{\{\|y\|>\lambda\}}\left(1-\frac{\lambda}{\|y\|}\right)_+ y$ *is the block soft threshold operator in real space.*

**Proof of Lemma 6.** Observe that

$$\arg\min_x \left(\frac{1}{2}(x-y)^\top(x-y) + \lambda\|x\|\right) = \arg\min_x \left(\frac{1}{2}(x^\top x - 2x^\top y) + \lambda\|x\|\right).$$

To satisfy the Karush–Kuhn–Tucker (KKT) stability condition, the derivative of the above objective function with respect to $x$ must be equal to zero. If the derivative does not exist, the subdifferential must include zero. The derivative is $x - y + \lambda s_x$, where $s_x$ is the subdifferential of $\|x\|$ at $x$.

If $x \neq 0$, $s_x = x/\|x\|$ and the KKT condition gives

$$x(1+\lambda/\|x\|) = y.$$

Compute the $\|y\|$ in the preceding equation and solve for $\|x\|$. Plugging it back into the equation gives us,

$$x = (1-\lambda/\|y\|)y.$$

The condition $x \neq 0$ is equivalent to $\|y\| > \lambda$. On the other hand, $x = 0$ is equivalent to $0 \in -y + \lambda s_x$, or $y \in \lambda s_x$. In this case $s_x = \{z \in \mathbb{R}^m | \|z\| \le 1\}$. Therefore, $\|y\|^2 \le \lambda^2$ which completes the proof. □

**Proof of Theorem 1.**

1) $\beta$-update.

Consider the objective function for $\beta$ in (9). After removing the constant terms with respect to $\beta$, with the help of Lemma 1, we have

$$\begin{aligned}[\beta^{\text{new}}] &:= \arg\min_\beta \left(f(\beta) + \frac{\rho}{2}([\beta]-[\gamma]+[U])^\top([\beta]-[\gamma]+[U])\right)\\ &= \arg\min_\beta \left(\frac{1}{2n}([\beta]^\top[\tilde{X}_{1:n}][\tilde{X}_{1:n}]^\top[\beta] - 2[\beta]^\top[\tilde{X}_{1:n}]Y) + \frac{\rho}{2}\{[\beta]^\top[\beta] - 2[\beta]^\top([\gamma]-[U])\}\right).\end{aligned}$$

Differentiate with respect to $\beta$, and set the derivative equal to zero to satisfy the KKT conditions. The result is:

$$n^{-1}[\tilde{X}_{1:n}][\tilde{X}_{1:n}]^\top[\beta] - n^{-1}[\tilde{X}_{1:n}]Y + \rho([\beta] - ([\gamma]-[U])) = 0.$$

Solve for $\beta$ which completes the derivation. Note that the result is similar to the functional ridge regression.

2) $\gamma$-update.

Similarly, if we remove the constant terms with respect to $\gamma$ and expand the objective function for $\gamma$, we have

$$\begin{aligned}[\gamma^{\text{new}}] &:= \arg\min_\gamma \left(g(\gamma) + \frac{\rho}{2}([\beta^{\text{new}}]-[\gamma]+[U])^\top([\beta^{\text{new}}]-[\gamma]+[U])\right)\\ &= \arg\min_\gamma \left(\sum_{j=1}^{p}\{\lambda([\gamma^j]^\top[\gamma^j])^{\frac{1}{2}} + \frac{\rho}{2}([\gamma^j] - ([(\beta^j)^{\text{new}}]+[U^j]))^\top([\gamma^j] - ([(\beta^j)^{\text{new}}]+[U^j]))\}\right).\end{aligned}$$

Note that the objective function is now additive which allows us to optimize $\gamma$ for each $\gamma^j$, $j = 1\ldots,p$. Thus, the above optimization is equivalent to

$$[(\gamma^j)^{\text{new}}] := \arg\min_{\gamma^j}\left(\lambda([\gamma^j]^\top[\gamma^j])^{\frac{1}{2}} + \frac{\rho}{2}([\gamma^j] - ([(\beta^j)^{\text{new}}]+[U^j]))^\top([\gamma^j] - ([(\beta^j)^{\text{new}}]+[U^j]))\right),$$

for $j = 1,\ldots p$. Applying Lemma 6 completes the proof. □

**Lemma 7** *For $x, y \in \mathbb{R}^m$ where $y$ is known and $a, b$ are constants*

$$\arg\min_x \left( \frac{1}{2}(x-y)^\top (x-y) + a(x^\top x)^{\frac{1}{2}} + \frac{b}{2} x^\top x \right) = \frac{1}{b+1} S_a(y).$$

**Proof of Lemma 7.**

The proof is similar to that of lemma 6. The only difference is the derivative of the objective function. It is $x - y + as + bx$, where $s$ is the subdifferential. The rest of the proof is straightforward. If $x \neq 0$ we see that $x(1 + b + \frac{a}{\|x\|}) = y$. Taking norm $\|.\|$ from both sides, solving for $\|x\|$, and plugging it back, we would have $x = (\frac{1}{1+b})(1 - \frac{a}{\|y\|})y$. Note that this is only possible when $\|x\| > 0$, which means $\|y\| > a$. If $x = 0$, it results in $0 \in -y + as$, or $y \in as$. Since in this case $s = \{[Z] | Z \in \mathbb{R}^m \& \|Z\| \leq 1\}$, $\|y\| \leq a$, which completes the derivation above. □

**Proof of Theorem 2.** The proof is a straightforward result from the combination of Theorem 1 and Lemma 7. □

**Lemma 8** *Assume that $\Gamma_{XX}$ is a positive definite operator and when $n$ approaches infinity, $\lambda_n$ approaches zero slower than the rate at which $\sqrt{n}$ approaches infinity. Then, $\|(\hat{\Gamma}_{XX} + \lambda_n I)^{-1}\Gamma_{XX} - (\Gamma_{XX} + \lambda_n I)^{-1}\Gamma_{XX}\|_{\mathcal{H}} = O_p(\lambda_n^{-1} n^{-1/2})$, and $\|(\hat{\Gamma}_{XX} + \lambda_n I)^{-1}\hat{\Gamma}_{XX} - (\Gamma_{XX} + \lambda_n I)^{-1}\Gamma_{XX}\|_{\mathcal{H}} = O_p(\lambda_n^{-1} n^{-1/2})$, where $\|\cdot\|_{\mathcal{H}}$ is the operator norm.*

**Proof of Lemma 8.** Note that $\Gamma_{XX}(\Gamma_{XX} + \lambda_n I) = I - \lambda_n(\Gamma_{XX} + \lambda_n I)^{-1}$ and $(\hat{\Gamma}_{XX} + \lambda_n I)\hat{\Gamma}_{XX} = I - \lambda_n(\hat{\Gamma}_{XX} + \lambda_n I)^{-1}$. Therefore,

$$(\hat{\Gamma}_{XX} + \lambda_n I)^{-1} - (\Gamma_{XX} + \lambda_n I)^{-1} = (\hat{\Gamma}_{XX} + \lambda_n I)^{-1}(\Gamma_{XX} - \hat{\Gamma}_{XX})(\Gamma_{XX} + \lambda_n I)^{-1}.$$

To be specific, if we add and subtract $\lambda_n(\hat{\Gamma}_{XX} + \lambda_n I)^{-1}(\Gamma_{XX} + \lambda_n I)^{-1}$ in the left-hand side of the above equation, we can easily derive the right-hand side of the equation. In addition, we have

$$\begin{aligned}
&(\hat{\Gamma}_{XX} + \lambda_n I)^{-1}\Gamma_{XX} - (\Gamma_{XX} + \lambda_n I)^{-1}\Gamma_{XX} \\
&= (\hat{\Gamma}_{XX} + \lambda_n I)^{-1}(\Gamma_{XX} - \hat{\Gamma}_{XX})(\Gamma_{XX} + \lambda_n I)^{-1}\Gamma_{XX}.
\end{aligned} \tag{24}$$

Note that $(\hat{\Gamma}_{XX} + \lambda_n I)^{-1} = (\Gamma_{XX} + O_p(n^{-1/2}) + \lambda_n I)^{-1}$ by Lemma 4. Thus, its norm is $\|(\hat{\Gamma}_{XX} + \lambda_n I)^{-1}\|_{\mathcal{H}} = O_p(\lambda_n^{-1})$. By Lemma 4, $\|(\Gamma_{XX} - \hat{\Gamma}_{XX})\|_{\mathcal{H}} = O_p(n^{-1/2})$. The norm of product of the last two parentheses is bounded by 1. Hence, $\|(\hat{\Gamma}_{XX} + \lambda_n I)^{-1}\Gamma_{XX} - (\Gamma_{XX} + \lambda_n I)^{-1}\Gamma_{XX}\|_{\mathcal{H}} = O_p(\lambda_n^{-1} n^{-1/2})$.

For the second convergence rate, note that

$$\begin{aligned}
&(\hat{\Gamma}_{XX} + \lambda_n I)^{-1}\hat{\Gamma}_{XX} - (\hat{\Gamma}_{XX} + \lambda_n I)^{-1}\Gamma_{XX} \\
&= (\hat{\Gamma}_{XX} + \lambda_n I)^{-1}(\hat{\Gamma}_{XX} - \Gamma_{XX}) = O_p(\lambda_n^{-1} n^{-1/2}).
\end{aligned}$$

Therefore,

$$\begin{aligned}
&\|(\hat{\Gamma}_{XX} + \lambda_n I)^{-1}\hat{\Gamma}_{XX} - (\Gamma_{XX} + \lambda_n I)^{-1}\Gamma_{XX}\|_{\mathcal{H}} \\
&\leq \|(\hat{\Gamma}_{XX} + \lambda_n I)^{-1}\hat{\Gamma}_{XX} - (\hat{\Gamma}_{XX} + \lambda_n I)^{-1}\Gamma_{XX}\|_{\mathcal{H}} \\
&+ \|(\hat{\Gamma}_{XX} + \lambda_n I)^{-1}\Gamma_{XX} - (\Gamma_{XX} + \lambda_n I)^{-1}\Gamma_{XX}\|_{\mathcal{H}} \\
&= O_p(\lambda_n^{-1} n^{-1/2}).
\end{aligned}$$

□

**Proof of Lemma 5.**

The following proof is similar to the proof mentioned in [1] which considers a different penalty term that is square of the group LASSO penalty. Then, they proved the consistency by stating that the solution path of the group LASSO will be the same. Instead, we consider a different optimization problem $\tilde{M}_n(.)$ proposed below, which directly leads to the consistency of multivariate functional group LASSO.

Denote $\tilde{\beta}_n^J$ as the unique minimizer of the following objective function.

$$\tilde{M}_n(\alpha) = \frac{1}{2}\hat{\Gamma}_{YY} - \hat{\Gamma}_{YX^J}(\alpha) + \frac{1}{2}\langle \alpha, \hat{\Gamma}_{X^J X^J}(\alpha)\rangle + \frac{\lambda_n}{2}\sum_{j\in J}\frac{\|\alpha^j\|^2_{\mathcal{H}^j}}{\|\beta^j\|_{\mathcal{H}^j}}, \quad \alpha \in \mathcal{H},$$

where $\beta^j$ is the $j$-th functional component of $\beta^J$ in the population model. $\tilde{\beta}_n^J$ has a closed-form solution similar to the solution of a functional predictor ridge regression

$$\tilde{\beta}_n^J = (\hat{\Gamma}_{X^J X^J} + \lambda_n D)^{-1}(\hat{\Gamma}_{X^J Y}),$$

where $D$ is a diagonal operator, $diag((\cdot)/\|\beta^j\|)$. We can replace $\hat{\Gamma}_{X^J Y}$ by the following expression, after adding and subtracting $\hat{\Gamma}_{X^J X^J}(\beta^J)$.

$$\tilde{\beta}_n^J = (\hat{\Gamma}_{X^J X^J} + \lambda_n D)^{-1}(\hat{\Gamma}_{X^J X^J}\beta^J + \hat{\Gamma}_{X\epsilon}), \tag{25}$$

where $\hat{\Gamma}_{X\epsilon}$ is the empirical covariance operator between observed functional data $X$ and the population error, $\epsilon = Y - \langle X, \beta\rangle = Y - \langle X^J, \beta^J\rangle$. $D$ is a self-adjoint operator, and $\|\beta^j\|_{\mathcal{H}} \neq 0$ for all $j \in J$ by the definition of the population active set $J$. This means there are positive constants $D_{\min} = 1/\max_{j\in J}\|\beta^j\|_{\mathcal{H}}$ and $D_{\max} = 1/\min_{j\in J}\|\beta^j\|_{\mathcal{H}}$ such that $D_{\max} I \succcurlyeq D \succcurlyeq D_{\min} I$. The closed-form solution (25) can be broken down into multiple terms. One of the terms is

$$(\hat{\Gamma}_{X^J X^J} + \lambda_n D)^{-1}(\hat{\Gamma}_{X\epsilon}). \tag{26}$$

Applying the same technique in the proof of Lemma 8 and using the result of Lemma 4, we can see that $\|\hat{\Gamma}_{X^J X^J} + \lambda_n D^{-1}\|_{\mathcal{H}} \leq D_{\min}^{-1}\lambda_n^{-1}$, and

$$(\hat{\Gamma}_{X^J X^J} + \lambda_n D)^{-1}(\hat{\Gamma}_{X\epsilon}) = O_p(n^{-1/2}\lambda_n^{-1}).$$

Hence, we have

$$\begin{aligned}
\tilde{\beta}_n^J - \beta^J &= (\hat{\Gamma}_{X^J X^J} + \lambda_n D)^{-1}(\hat{\Gamma}_{X^J X^J}\beta^J + \hat{\Gamma}_{X\epsilon}) - \beta^J \\
&= (\hat{\Gamma}_{X^J X^J} + \lambda_n D)^{-1}(\hat{\Gamma}_{X^J X^J}\beta^J) - (\Gamma_{X^J X^J} + \lambda_n D)^{-1}\Gamma_{X^J X^J}\beta^J \\
&\quad + (\Gamma_{X^J X^J} + \lambda_n D)^{-1}\Gamma_{X^J X^J}\beta^J - \beta^J + O_p(n^{-1/2}\lambda_n^{-1})
\end{aligned} \tag{27}$$

The first two terms of the last equation in (27) is $O_p(n^{-1/2}\lambda_n^{-1})$ by Lemma 8. By using $(\Gamma_{X^J X^J} + \lambda_n D)^{-1}\Gamma_{X^J X^J} = I - \lambda_n(\Gamma_{X^J X^J} + \lambda_n D)^{-1}D$, we can simplify the third and fourth terms of (27) as

$$(\Gamma_{X^J X^J} + \lambda_n D)^{-1}\Gamma_{X^J X^J}\beta^J - \beta^J = (-\lambda_n(\Gamma_{X^J X^J} + \lambda_n D)^{-1}D)\beta^J. \tag{28}$$

Consequently, we have

$$\tilde{\beta}_n^J - \beta^J = (-\lambda_n(\Gamma_{X^J X^J} + \lambda_n D)^{-1}D)\beta^J + O_p(n^{-1/2}\lambda_n^{-1}). \tag{29}$$

Now, we show the norm of $\lambda_n(\Gamma_{X^J X^J} + \lambda_n D)^{-1}D$ is $O_p(\sqrt{\lambda_n} + n^{-1/2}\lambda_n^{-1})$. Let $h^J \in \mathcal{H}^J$ be the element in the assumption such that $\beta^J = \Gamma_{X^J X^J}^{1/2}h^J$. Then,

$$\begin{aligned}
\|\lambda_n(\Gamma_{X^J X^J} + \lambda_n D)^{-1}D\beta^J\|^2_{\mathcal{H}^J} &= \lambda_n^2\langle \beta^J, D(\Gamma_{X^J X^J} + \lambda_n D)^{-2}D\beta^J\rangle_{\mathcal{H}^J} \\
&\leq \lambda_n^2 D_{\max}^2\langle \beta^J, (\Gamma_{X^J X^J} + \lambda_n D_{\min} I)^{-2}\beta^J\rangle_{\mathcal{H}^J} \\
&\leq \lambda_n D_{\max}^2 D_{\min}^{-1}\langle \beta^J, (\Gamma_{X^J X^J} + \lambda_n D_{\min} I)^{-1}\beta^J\rangle_{\mathcal{H}^J} \\
&= \lambda_n D_{\max}^2 D_{\min}^{-1}\langle \Gamma_{X^J X^J}^{1/2}h^J, (\Gamma_{X^J X^J} + \lambda_n D_{\min} I)^{-1}\Gamma_{X^J X^J}^{1/2}h^J\rangle_{\mathcal{H}^J} \\
&\leq \lambda_n D_{\max}^2 D_{\min}^{-1}\|h^J\|^2_{\mathcal{H}}.
\end{aligned}$$

The third line of the above equation is valid because $\|\Gamma_{X^J X^J} + \lambda_n D_{\min} I\|_{\mathcal{H}^J} \geq \lambda_n D_{\min}$. Combining the results above, we have

$$\|\tilde{\beta}_n^J - \beta^J\|_{\mathcal{H}} = O_p(\sqrt{\lambda_n} + n^{-1/2}\lambda_n^{-1}).$$

Now, let's compare $\tilde{\beta}_n^J$ and $\beta_n^J$ where $\beta_n^J$ is the solution to the optimization problem of $M_n(\alpha)$. Consider the following equation.

$$M_n(\alpha) - \tilde{M}_n(\alpha) = \lambda_n \sum_{j \in J} \left( \|\alpha^j\|_{\mathcal{H}^j} - \frac{\|\alpha^j\|^2_{\mathcal{H}^j}}{2\|\beta^j\|_{\mathcal{H}^j}} \right). \tag{30}$$

The partial Fréchet derivative of the equation (30) with respect to $\alpha^i$ for an $i \in J$ is

$$D_{\alpha^i}(M_n(\alpha) - \tilde{M}_n(\alpha)) = \lambda_n \left( \frac{\langle \alpha^i, \cdot \rangle_{\mathcal{H}^i}}{\|\alpha^i\|_{\mathcal{H}^i}} - \frac{\langle \alpha^i, \cdot \rangle_{\mathcal{H}^i}}{\|\beta^i\|_{\mathcal{H}^i}} \right). \tag{31}$$

Since $\beta^J$ are nonzero, (31) is continuously differentiable around $\beta^J$, and $D_{\alpha^i}\tilde{M}_n(\tilde{\beta}_n^J)) = 0$, we have

$$\|D_{\alpha^i} M_n(\tilde{\beta}_n^J)) - 0\| = \lambda_n \left\| \frac{\langle \tilde{\beta}_n^i, \cdot \rangle_{\mathcal{H}^i}}{\|\tilde{\beta}_n^i\|_{\mathcal{H}^i}} - \frac{\langle \tilde{\beta}_n^i, \cdot \rangle_{\mathcal{H}^i}}{\|\beta^i\|_{\mathcal{H}^i}} \right\|,$$

where the $\|\cdot\|$ is the operator norm. In addition, since $\beta^i \neq 0$ for $i \in J$, it can be easily shown that

$$\|D_{\alpha^i} M_n(\tilde{\beta}_n^J)) - 0\|_{\mathcal{H}^i} \leq C\lambda_n \|\beta^J - \tilde{\beta}_n^J\|_{\mathcal{H}^J},$$

for some constant $C > 0$. Thus, we have

$$\|D_{\alpha^i} M_n(\tilde{\beta}_n^J))\|_{\mathcal{H}^i} = \lambda_n O_p(\lambda_n^{1/2} + n^{-1/2}\lambda_n^{-1}). \tag{32}$$

Now, since $M_n$ is strictly convex near the true $\beta^J$, its second-order Fréchet derivative has a lower bound. Consequently, we have

$$M_n(\alpha^J) \geq M_n(\tilde{\beta}_n^J) + \langle D_{\alpha^J} M_n(\tilde{\beta}_n^J), (\alpha^J - \tilde{\beta}_n^J) \rangle_{\mathcal{H}^J} + C'\lambda_n \|\alpha^J - \tilde{\beta}_n^J\|^2_{\mathcal{H}^J},$$

for some $C' > 0$. Suppose that $\alpha^J$ is near $\tilde{\beta}_n^J$ and let $\eta_n = \|\alpha^J - \tilde{\beta}_n^J\|^2_{\mathcal{H}^J}$ which tends to zero. Subsequently, we can rewrite the lower bound such that

$$M_n(\alpha^J) \geq M_n(\tilde{\beta}_n^J) + \eta_n \lambda_n O_p(\sqrt{\lambda_n} + n^{-1/2}\lambda_n^{-1}) + C'\lambda_n \eta_n^2, \tag{33}$$

If the last term is tending to zero slower than the second term, we can conclude that all minima of $M_n(\cdot)$ are inside the ball $\{\alpha^J : \|\alpha^J - \tilde{\beta}_n^J\|^2_{\mathcal{H}^J} < \eta\}$ with probability tending to one. This is because $M_n(\cdot)$, on the edge of the ball, takes values greater the ones inside the ball. i.e., the global minimum of $M_n(\cdot)$ is at most $\eta_n$ away from $\tilde{\beta}_n^J$. Thus, the necessary condition for the proof is $\eta_n \lambda_n^{3/2} = o(\lambda_n \eta_n^2)$ and $n^{-1/2}\eta_n = o(\lambda_n \eta_n^2)$. Alltogether, we have the consistency results if $\eta_n$ converges to zero slower than $\lambda_n^{1/2} + n^{-1/2}\lambda_n^{-1}$. □

**Proof of Theorem 3**. We rewrite the multivariate functional group LASSO objective function (3) as,

$$\hat{M}_n(\alpha) = \frac{1}{2}\hat{\Gamma}_{YY} - \hat{\Gamma}_{YX}\alpha + \frac{1}{2}\langle \alpha, \hat{\Gamma}_{XX}\alpha \rangle_{\mathcal{H}} + \lambda_n \sum_{j=1}^{p} \|\alpha^j\|_{\mathcal{H}^j}.$$

Denote a minimizer of $\hat{M}_n(\cdot)$ by $\hat{\beta}_n$. Since it is a convex function, it has a unique minimizer. In addition, if $\lambda_n$ goes to zero, the objective function converges to the regression problem without the penalty whose unique minimizer is $\beta$. Thus, it is easy to see that $\hat{J} = \{j : \hat{\beta}_n^j(\cdot) \neq 0\}$ converges to $J$ via the M-estimation theory. See [2] and [3].

Now, we extend $\beta_n^J$ in Lemma 5 with zero functions as $\beta_n^i$ for $i \in J^c$, name it $\beta_n \in \mathcal{H}$. Note that, it is a consistent estimator of $\beta$ by Lemma 5. Since both of the $M_n(\cdot)$ and $\hat{M}_n(\cdot)$ have unique minimizers and the $\beta_n$ is a consistent estimator of $\beta$, the consistency of $\hat{\beta}_n$ can be shown, if we can show that $\beta_n$ satisfies the optimal conditions for $\hat{M}_n(\cdot)$ with a probability tending to one. The (asymptotically) optimal conditions of $\hat{M}_n(\cdot)$ are

$$\begin{cases} \|\hat{\Gamma}_{X^iX}\alpha - \hat{\Gamma}_{X^iY}\|_{\mathcal{H}^i} \leq \lambda_n & i \notin J \\ \langle \hat{\Gamma}_{X^jX}\alpha, \cdot\rangle_{\mathcal{H}^j} - \hat{\Gamma}_{YX^j}(\cdot) = -\frac{\lambda_n}{\|\alpha^j\|_{\mathcal{H}^j}}\langle \alpha^j, \cdot\rangle_{\mathcal{H}^j} & j \in J. \end{cases}$$

The second equation is immediately satisfied with $\alpha = \beta_n$, since it satisfies the KKT condition for $M_n(\cdot)$. We focus on the above inequality of the optimal condition. The first derivative condition for minimizing $M_n(\cdot)$ implies that $\beta_n^J$ should justify the following equation

$$-\hat{\Gamma}_{YX^J}(\cdot) + \langle \hat{\Gamma}_{X^JX^J}\beta_n^J, \cdot\rangle_{\mathcal{H}^J} + \lambda_n \sum_{j \in J} \frac{\langle \beta_n^j, \cdot\rangle_{\mathcal{H}^j}}{\|\beta_n^j\|_{\mathcal{H}^j}} = 0.$$

Define $D_n$ be an operator from $\mathcal{H}^J$ to $\mathcal{H}^J$ such that $D_n(\alpha^J) = diag(\alpha^j/\|\beta_n^j\|)$ for $j \in J$. We rewrite the above equation as

$$-\hat{\Gamma}_{YX^J}(\cdot) + \langle (\hat{\Gamma}_{X^JX^J} + \lambda_n D_n)\beta_n^J, \cdot\rangle_{\mathcal{H}^J} = 0.$$

In addition, note that

$$\hat{\Gamma}_{YX^J}(\cdot) = \langle \hat{\Gamma}_{X^JY}, \cdot\rangle_{\mathcal{H}^J} = \langle \hat{\Gamma}_{X^JX^J}\beta^J + \hat{\Gamma}_{X\epsilon}, \cdot\rangle_{\mathcal{H}^J}.$$

Thus, we have

$$\langle \beta_n^J, \cdot\rangle = \langle (\hat{\Gamma}_{X^JX^J} + \lambda_n D_n)^{-1}(\hat{\Gamma}_{X^JX^J}\beta^J + \hat{\Gamma}_{X^J\epsilon}), \cdot\rangle_{\mathcal{H}^J}.$$

Furthermore, by using a similar technique used in (28),

$$(\hat{\Gamma}_{X^JX^J} + \lambda_n D_n)^{-1}\hat{\Gamma}_{X^JX^J}\beta^J = \beta^J - (\hat{\Gamma}_{X^JX^J} + \lambda_n D_n)^{-1}\lambda_n D_n \beta^J.$$

Thus, for an $i \in J^c$:

$$\begin{aligned} \hat{\Gamma}_{X^iY} - \hat{\Gamma}_{X^iX^J}\beta_n^J &= \hat{\Gamma}_{X^iY} - \hat{\Gamma}_{X^iX^J}\beta^J + \lambda_n\hat{\Gamma}_{X^iX^J}(\hat{\Gamma}_{X^JX^J} + \lambda_n D_n)^{-1}D_n\beta^J \\ &\quad - \hat{\Gamma}_{X^iX^J}(\hat{\Gamma}_{X^JX^J} + \lambda_n D_n)^{-1}\hat{\Gamma}_{X^J\epsilon} \\ &= \lambda_n\hat{\Gamma}_{X^iX^J}(\hat{\Gamma}_{X^JX^J} + \lambda_n D_n)^{-1}D_n\beta^J + \hat{\Gamma}_{X^i\epsilon} \\ &\quad - \hat{\Gamma}_{X^iX^J}(\hat{\Gamma}_{X^JX^J} + \lambda_n D_n)^{-1}\hat{\Gamma}_{X^J\epsilon}, \end{aligned}$$

by using the fact that $\hat{\Gamma}_{X^iY} - \hat{\Gamma}_{X^iX^J}(\beta^J) = \hat{\Gamma}_{X^i\epsilon}$. At this point, the formulation has a similar form, derived in Theorem 11 of [1]. Furthermore, Lemma 5 satisfies the condition that is necessary to derive the rest of the proof so that they can be derived similarly. □

# 3 Rregions of Interests

**List of regions of interests**: The following are the lists of the regions of interest of the human brain used in the application section 8. The atlas labels of the human brain and full names can be found at Atlas Label.

The list of the regions of interest associated with the active set of MFG-Lasso when the response value is IQ score:
"Frontal–Mid–Orb–L", "Frontal–Mid–Orb–R", "Frontal–Inf–Oper–L", "Frontal–Inf–Oper–R","Frontal–Inf–Tri–L", "Frontal–Inf–Tri–R", "Frontal–Inf–Orb–L","Frontal–Inf–Orb–R", "Rolandic–Oper–R", "Supp–Motor–Area–L", "Olfactory–L", "Olfactory–R", "Frontal–Sup–Medial–L","Frontal–Med–Orb–L", "Frontal–Med–Orb–R", "Rectus–L", "Cingulum–Ant–L", "Cingulum–Post–L", "Cingulum–Post–R", "Amygdala–L", "Amygdala–R", "Calcarine–L", "Calcarine–R", "Cuneus–L", "Cuneus–R", "Lingual–L", "Occipital–Sup–L", "Occipital–Sup–R", "Occipital–Mid–R", "Occipital–Inf–L", "Occipital–Inf–R", "Parietal–Sup–L", "Parietal–Inf–R", "SupraMarginal–L", "SupraMarginal–R", "Angular–L", "Angular–R", "Precuneus–L", "Paracentral–Lobule–L", "Paracentral–Lobule–R","Putamen–L", "Pallidum–R", "Heschl–R", "Temporal–Sup–L", "Temporal–Pole–Mid–L", "Cerebellum–3–L", "Cerebellum–3–R", "Vermis–1–2", "Vermis–3", "Vermis–4–5", "Vermis–6", "Vermis–9", "Vermis–10".

The list of the regions of interest associated with the active set of MFG-Lasso when the response value is Verbal IQ:
"Frontal–Sup–R", "Frontal–Mid–Orb–L","Frontal–Mid–Orb–R", "Frontal–Inf–Oper–R","Frontal–Inf–Tri–L","Frontal–Inf–Tri–R", "Frontal–Inf–Orb–L","Frontal–Inf–Orb–R","Rolandic–Oper–R", "Supp–Motor–Area–L","Olfactory–L", "Frontal–Sup–Medial–L" "Frontal–Med–Orb–L","Frontal–Med–Orb–R","Rectus–L", "Cingulum–Ant–L", "Cingulum–Post–L", "Cingulum–Post–R", "Amygdala–L", "Amygdala–R", "Calcarine–L", "Calcarine–R", "Cuneus–L", "Cuneus–R", "Occipital–Sup–L", "Parietal–Sup–L", "Parietal–Sup–R", "Parietal–Inf–L", "Parietal–Inf–R", "SupraMarginal–L", "SupraMarginal–R", "Angular–L", "Precuneus–L", "Precuneus–R", "Paracentral–Lobule–L","Paracentral–Lobule–R" "Putamen–L", "Pallidum–R", "Heschl–R", "Temporal–Sup–L", "Temporal–Pole–Mid–L","Cerebellum–3–L", "Vermis–1–2", "Vermis–3", "Vermis–4–5", "Vermis–6", "Vermis–9", "Vermis–10", .

The list of the regions of interest associated with the active set of MFG-Lasso when the response value is Performance IQ:
"Frontal–Sup–Orb–L","Frontal–Mid–Orb–L","Frontal–Mid–Orb–R", "Frontal–Inf–Oper–L","Frontal–Inf–Oper–R","Frontal–Inf–Tri–L", "Frontal–Inf–Tri–R","Frontal–Inf–Orb–L","Frontal–Inf–Orb–R", "Rolandic–Oper–R", "Supp–Motor–Area–L","Olfactory–L", "Olfactory–R", "Frontal–Sup–Medial–L","Frontal–Sup–Medial–R" "Frontal–Med–Orb–L","Frontal–Med–Orb–R","Rectus–L", "Insula–R", "Cingulum–Ant–L", "Cingulum–Mid–L", "Cingulum–Post–L", "Cingulum–Post–R", "ParaHippocampal–L", "ParaHippocampal–R","Amygdala–L", "Amygdala–R", "Calcarine–L", "Calcarine–R", "Cuneus–L", "Cuneus–R", "Lingual–L", "Occipital–Sup–L", "Occipital–Sup–R", "Occipital–Mid–L", "Occipital–Mid–R", "Occipital–Inf–L", "Occipital–Inf–R", "Postcentral–L", "Postcentral–R", "Parietal–Sup–L", "Parietal–Sup–R", "Parietal–Inf–L", "Parietal–Inf–R", "SupraMarginal–L", "SupraMarginal–R", "Angular–L", "Angular–R", "Precuneus–L", "Precuneus–R", "Paracentral–Lobule–L" "Paracentral–Lobule–R","Caudate–L", "Putamen–L", "Pallidum–R", "Thalamus–L", "Heschl–L", "Heschl–R", "Temporal–Sup–L", "Temporal–Pole–Sup–L", "Temporal–Pole–Sup–R","Temporal–Mid–L", "Temporal–Pole–Mid–L", "Temporal–Pole–Mid–R","Cerebellum–3–L", "Cerebellum–3–R", "Cerebellum–4–5–R", "Cerebellum–6–L", "Cerebellum–6–R", "Vermis–1–2", "Vermis–3", "Vermis–4–5", "Vermis–6", "Vermis–7", "Vermis–9", "Vermis–10".

The list of the regions of interest associated with the active set of MFG-Lasso when the response value is ADHD score:
"Frontal–Mid–L", "Frontal–Mid–Orb–L","Frontal–Mid–Orb–R", "Frontal–Inf–Oper–L","Frontal–Inf–Oper–R","Frontal–Inf–Tri–L", "Frontal–Inf–Orb–L","Frontal–Inf–Orb–R","Supp–Motor–Area–L", "Olfactory–L", "Frontal–Sup–Medial–L","Frontal–Sup–Medial–R" "Frontal–Med–Orb–L","Rectus–L", "Cingulum–Ant–L", "Cingulum–Post–L", "ParaHippocampal–R","Amygdala–L", "Calcarine–L", "Cuneus–L", "Cuneus–R", "Occipital–Inf–L", "Occipital–Inf–R", "Parietal–Sup–L", "Parietal–Inf–L", "SupraMarginal–L", "SupraMarginal–R", "Angular–L", "Angular–R", "Precuneus–L", "Paracentral–Lobule–L","Paracentral–Lobule–R","Putamen–L", "Heschl–R", "Temporal–Sup–L", "Temporal–Pole–Sup–R", "Temporal–Pole–Mid–L","Cerebellum–9–L", "Vermis–1–2", "Vermis–4–5", "Vermis–10".

The list of the regions of interest associated with the active set of MFG-Lasso when the response value is ADHD Inattentive:
"Frontal–Mid–Orb–L","Frontal–Mid–Orb–R","Frontal–Inf–Oper–L", "Frontal–Inf–Oper–R","Frontal–Inf–Tri–L","Frontal–Inf–Orb–L", "Frontal–Inf–Orb–R","Supp–Motor–Area–L","Frontal–Sup–Medial–L" "Frontal–Sup–Medial–R","Frontal–Med–Orb–L","Rectus–L", "Cingulum–Ant–L", "Cingulum–Post–L", "Cingulum–Post–R", "ParaHippocampal–R","Amygdala–L", "Calcarine–L", "Cuneus–L", "Cuneus–R", "Lingual–L", "Occipital–Inf–L", "Occipital–Inf–R", "Parietal–Sup–L", "Parietal–Inf–L", "SupraMarginal–L", "SupraMarginal–R", "Angular–L", "Angular–R", "Precuneus–L", "Precuneus–R", "Paracentral–Lobule–L","Paracentral–Lobule–R" "Heschl–R", "Temporal–Sup–L", "Temporal–Pole–Sup–R", "Temporal–Pole–Mid–L","Cerebellum–4–5–R", "Vermis–1–2", "Vermis–4–5", "Vermis–10".

The list of the regions of interest associated with the active set of MFG-Lasso when the

response value is ADHD Hyper/Impulsive:

"Frontal–Mid–Orb–L","Frontal–Mid–Orb–R","Frontal–Inf–Oper–L", "Frontal–Inf–Oper–R","Frontal–Inf–Tri–L","Frontal–Inf–Orb–L", "Frontal–Inf–Orb–R","Rolandic–Oper–R", "Supp–Motor–Area–L", "Olfactory–L", "Frontal–Sup–Medial–L","Frontal–Sup–Medial–R" "Frontal–Med–Orb–L","Frontal–Med–Orb–R","Rectus–L", "Rectus–R", "Cingulum–Ant–L", "Cingulum–Mid–L", "Cingulum–Post–L", "ParaHippocampal–R","Amygdala–L", "Amygdala–R", "Calcarine–L", "Cuneus–L", "Cuneus–R", "Occipital–Sup–R", "Occipital–Mid–R", "Occipital–Inf–L", "Occipital–Inf–R", "Parietal–Sup–L", "Parietal–Inf–L", "Parietal–Inf–R", "SupraMarginal–L", "SupraMarginal–R", "Angular–L", "Angular–R", "Putamen–L", "Pallidum–R", "Heschl–L", "Heschl–R", "Temporal–Sup–L", "Temporal–Pole–Sup–R", "Temporal–Pole–Mid–L","Temporal–Pole–Mid–R","Cerebellum–3–R", "Cerebellum–4–5–R", "Cerebellum–9–L", "Vermis–1–2", "Vermis–3", "Vermis–4–5", "Vermis–6", "Vermis–7", "Vermis–10".

The list of the regions that are associated with IQ but not with ADHD by the MFG-Lasso:

"Frontal–Inf–Tri–R","Rolandic–Oper–R","Olfactory–R", "Frontal–Med–Orb–R","Cingulum–Post–R", "Amygdala–R", "Calcarine–R", "Lingual–L", "Occipital–Sup–L","Occipital–Sup–R", "Occipital–Mid–R","Parietal–Inf–R","Pallidum–R", "Cerebellum–3–L", "Cerebellum–3–R", "Vermis–3", "Vermis–6", "Vermis–9",

The list of the regions that are associated with ADHD but not with IQ by the MFG-Lasso:

"Frontal–Mid–L", "Frontal–Sup–Medial–R","ParaHippocampal–R","Parietal–Inf–L", "Temporal–Pole–Sup–R","Cerebellum–9–L".

# References


1. Bach FR. Consistency of the Group Lasso and Multiple Kernel Learning. Journal of Machine Learning Research. 2008;9:1179–1225.
2. Van der Vaart AW. Asymptotic statistics. Vol 3. Cambridge university press; 2000.
3. Knight K, Fu W. Asymptotics for lasso-type estimators. The Annals of statistics. 2000; p. 1356–1378.
