# Supplementary material for: Multivariate functional group sparse regression: Functional predictor selection
Source: PLoS One. 2022 Apr 7;17(4):e0265940. doi: 10.1371/journal.pone.0265940 (PMC8989243; doi:10.1371/journal.pone.0265940)
Supplement: S1 Table — (PDF) [file pone.0265940.s002.pdf]

S1 Table. Average test RMSE of different methods under different simulation scenarios when we have unbalanced time points for each observation. In each case, 100 random samples are used to compute the mean and standard deviation with parentheses.

Parameters		Methods				
σ	n	OLS	Ridge	MFG-LASSO	MFG-EN	Oracle
0.01	100	3.21 (0.63)	2.44 (0.45)	1.67 (0.35)	1.69 (0.35)	1.54 (0.38)
	200	2.08 (0.32)	1.73 (0.23)	1.42 (0.2)	1.43 (0.2)	1.38 (0.22)
	500	1.52 (0.18)	1.43 (0.15)	1.3 (0.16)	1.3 (0.15)	1.27 (0.15)
0.1	100	3.21 (0.63)	2.44 (0.45)	1.67 (0.35)	1.7 (0.34)	1.55 (0.39)
	200	2.09 (0.32)	1.74 (0.23)	1.43 (0.2)	1.44 (0.2)	1.39 (0.22)
	500	1.52 (0.18)	1.43 (0.15)	1.3 (0.16)	1.31 (0.15)	1.28 (0.15)
1	100	4.04 (0.79)	2.88 (0.53)	2.1 (0.38)	2.14 (0.39)	1.93 (0.38)
	200	2.7 (0.38)	2.18 (0.27)	1.81 (0.24)	1.82 (0.23)	1.76 (0.24)
	500	1.94 (0.16)	1.82 (0.16)	1.67 (0.16)	1.67 (0.15)	1.63 (0.16)
