# Supplementary material for: Multivariate functional group sparse regression: Functional predictor selection
Source: PLoS One. 2022 Apr 7;17(4):e0265940. doi: 10.1371/journal.pone.0265940 (PMC8989243; doi:10.1371/journal.pone.0265940)
Supplement: S2 Table — (PDF) [file pone.0265940.s003.pdf]

S2 Table. Percentages of correct selection in the test set under various simulation scenarios when we have unbalanced time points for each observation. In each case, 100 random samples are used. In each sample, we count the correctly identified functional predictors for the active set of the size 3 and the inactive set of the size 16. Then, we compute the overall percentage out of 100 samples.

Parameters		Selection	Methods	
σ	n		MFG-LASSO	MFG-EN
0.01	100	Inactive	27	22
		Active	100	100
	200	Inactive	31	26
		Active	100	100
	500	Inactive	41	34
		Active	100	100
0.1	100	Inactive	26	22
		Active	100	100
	200	Inactive	32	25
		Active	100	100
	500	Inactive	41	34
		Active	100	100
1	100	Inactive	24	21
		Active	100	100
	200	Inactive	30	28
		Active	100	100
	500	Inactive	39	32
		Active	100	100
